# MiR-34a Targeting of Notch Ligand Delta-Like 1 Impairs CD15^+^/CD133^+^ Tumor-Propagating Cells and Supports Neural Differentiation in Medulloblastoma

**DOI:** 10.1371/journal.pone.0024584

**Published:** 2011-09-12

**Authors:** Pasqualino de Antonellis, Chiara Medaglia, Emilio Cusanelli, Immacolata Andolfo, Lucia Liguori, Gennaro De Vita, Marianeve Carotenuto, Annamaria Bello, Fabio Formiggini, Aldo Galeone, Giuseppe De Rosa, Antonella Virgilio, Immacolata Scognamiglio, Manuela Sciro, Giuseppe Basso, Johannes H. Schulte, Giuseppe Cinalli, Achille Iolascon, Massimo Zollo

**Affiliations:** 1 Centro di Ingegneria Genetica e Biotecnologia Avanzate (CEINGE), Naples, Italy; 2 Laboratory of Hematology–Oncology, Department of Pediatrics, University of Padova, Padua, Italy; 3 University Children's Hospital Essen, Essen, Germany; 4 Dipartimento di Chimica delle Sostanze Naturali, “Federico II” University of Naples, Naples, Italy; 5 Dipartimento di Chimica Farmaceutica e Tossicologia, “Federico II” University of Naples, Naples, Italy; 6 Struttura Complessa di Neurochirurgia, Ospedale Pediatrico Santobono–Pausilipon, Naples, Italy; 7 Dipartimento di Biochimica e Biotecnologie Mediche (DBBM), “Federico II” University of Naples, Naples, Italy; RMIT University, Australia

## Abstract

**Background:**

Through negative regulation of gene expression, microRNAs (miRNAs) can function as oncosuppressors in cancers, and can themselves show altered expression in various tumor types. Here, we have investigated medulloblastoma tumors (MBs), which arise from an early impairment of developmental processes in the cerebellum, where Notch signaling is involved in many of the cell-fate-determining stages. Notch regulates a subset of MB cells that have stem-cell-like properties and can promote tumor growth. On the basis of this evidence, we hypothesized that miRNAs targeting the Notch pathway can regulate these phenomena, and can be used in anti-cancer therapies.

**Methodology/Principal Findings:**

In a screening of potential targets within Notch signaling, miR-34a was seen to be a regulator of the Notch pathway through its targeting of Notch ligand Delta-like 1 (Dll1). Down-regulation of Dll1 expression by miR-34a negatively regulates cell proliferation, and induces apoptosis and neural differentiation in MB cells. Using an inducible tetracycline on-off model of miR-34a expression, we show that in Daoy MB cells, Dll1 is the first target that is regulated in MB, as compared to the other targets analyzed here: Cyclin D1, cMyc and CDK4. MiR-34a expression negatively affects CD133^+^/CD15^+^ tumor-propagating cells, then we assay through reverse-phase proteomic arrays, Akt and Stat3 signaling hypo-phosphorylation. Adenoviruses carrying the precursor miR-34a induce neurogenesis of tumor spheres derived from a genetic animal model of MB (Patch1^+/-^ p53^-/-^), thus providing further evidence that the miR-34a/Dll1 axis controls both *autonomous* and *non autonomous* signaling of Notch. *In vivo*, miR-34a overexpression carried by adenoviruses reduces tumor burden in cerebellum xenografts of athymic mice, thus demonstrating an anti-tumorigenic role of miR-34a *in vivo*.

**Conclusions/Significance:**

Despite advances in our understanding of the pathogenesis of MB, one-third of patients with MB remain incurable. Here, we show that stable nucleic-acid-lipid particles carrying mature miR-34a can target Dll1 *in vitro* and show equal effects to those of adenovirus miR-34a cell infection. Thus, this technology forms the basis for their therapeutic use for the delivery of miR-34a in brain-tumor treatment, with no signs of toxicity described to date in non-human primate trials.

## Introduction

Medulloblastoma (MB) is the most common malignant and highly invasive embryonal tumor in children. It originates in the cerebellum, and accounts for more than 25% of childhood cancer-related deaths [1]. MB can arise from granule-cell progenitors and neural stem cells (NSCs) of the cerebellum [2]. Pathways such as Notch and Sonic Hedgehog (Shh), which control cerebellum development, are crucially involved in MB tumorigenesis [3], [4].

MiRNAs are involved in virtually all biological processes, and several studies have demonstrated their roles in human tumorigenesis [5]. We and others have described several miRNAs that are involved in MB development, including miR-125b, miR-324-5p, miR-326 and miR-199b-5p [6], [7], [8]. MiR-199b-5p regulates the *Hes1* gene, a key effector of the Notch pathway, and inhibits proliferation and survival of MB CD133^+^ cancer-stem-cell populations.

The MiR-34 family is directly regulated by the transcription factor p53 [9], [10], [11], and all of the members of this family (miR-34a, mi-R34b and miR-34c) share high sequence similarities [12]. MiR-34a affects the typical p53 oncosuppressor activity, by inhibiting cell growth, inducing apoptosis and causing a senescence-like phenotype [13]. Several studies have confirmed that the miR-34 family is required for normal cell responses to DNA damage following irradiation *in vivo*. This evidence led to a model for the potential therapeutic use of miR-34 as a radio-sensitizing agent in p53-mutant breast cancer [14]. However, these effects are cell-type dependent, as miR-34a also supports cell proliferation in HeLa and MCF-7 cells [15]. Comparative expression analyses have shown that miR-34a is highest within the cerebellar cortex [15] and brain tissues [12], and that it acts as a tumor suppressor in gliomas, by targeting both E2F3 and MYCN, and by regulating cell-cycle and apoptosis genes. In gliomas, transfection of miR-34a down-regulates c-Met and CDK6, as also for Notch1 and Notch2, which suggests that miR-34a provides a therapeutic agent for brain tumors, through its targeting of multiple oncogenes [16]. Human-brain tumor-propagating cells (TPCs) [17], [18] express CD133 (CD133^+^) and are also CD15^+^ (also known as SSEA-1 or LeX), and they resemble neural progenitors, as they show clonogenic and multilineage differentiation capacity, and the ability to initiate tumors following orthotopic xenograft transplantation [19]. Ji et al. (2009) showed that in MiaPaCa2 pancreatic cancer cells, functional restoration of miR-34a down-regulates CD44^+^/CD133^+^ cells by inhibiting its downstream target genes Notch and Bcl-2, and impairs tumor-sphere growth *in vitro* and tumor formation *in vivo* [20].

The present study started with the hypothesis of additional miR-34a targets as key genes in Notch and Shh signaling. Given the crucial roles of these pathways in MB tumorigenesis and cancer-stem-cell maintenance, we investigated whether miR-34a can mediate the development of MB tumorigenesis. Our study shows that miR-34a is a key negative regulator of Notch ligand Delta-like 1 (Dll1) and influences Notch1 and Notch2 signaling in the cell in both an *autonomous* and *non autonomous* manner. Hence, miR-34a inhibits cell proliferation, enhances apoptosis, induces cell differentiation and further impairs TPC preservation *in vitro*. *In vivo*, we show miR-34a inhibition of tumor growth in orthotopic xenografts of athymic nude mice. Thus, we have established here a strong rationale for the development of miR-34a as a novel therapeutic agent against MB TPCs.

## Results

The Notch signaling pathway is known to be relevant in MB development, so we used target-prediction analyses to determine whether miR-34a has any role within Notch signaling. In doing so, we noted that several predicted targets of miR-34a are key genes of the Notch pathway: Dll1, Jagged1 (Jag1), Notch1 and Notch2, which represent two ligands and two receptors of the Notch pathway, respectively (Table S1). Then, using luciferase reporter assays, we investigated whether miR-34a effectively recognizes the 3’-UTR of these selected genes in MB cells.

**Figure 1:**
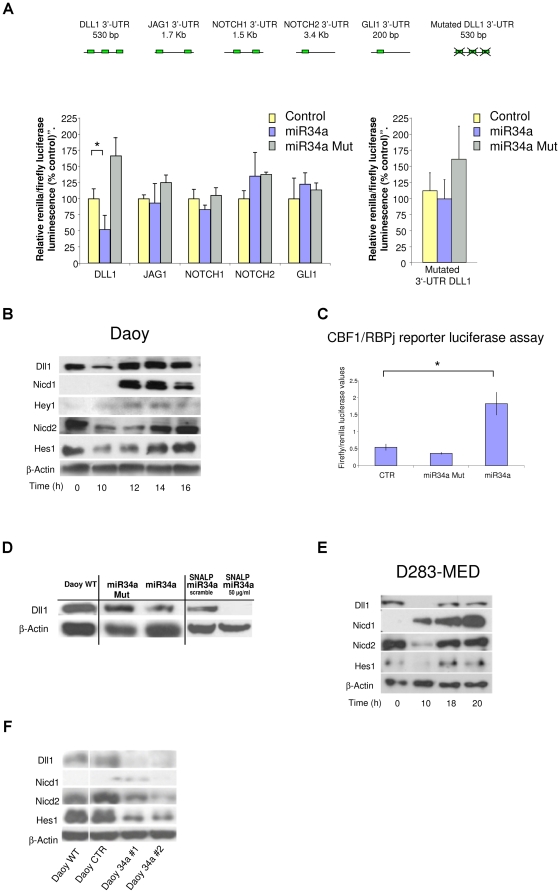
Direct recognition and validation of miR-34a target genes using a luciferase assay and time-course overexpression assays of miR-34a in MB Daoy and D283-MED cell lines. **A.** Top: Representative 3’-UTR diagram showing the predicted miR-34a binding sites in individual 3’-UTRs. Bottom left: Luciferase assay on Daoy cells co-transfected with individual 3’-UTR reporter constructs, the pGL3 control vector, and wild-type or seed-mutated miR-34a. The relative luciferase activities at 24 h from transfection are given, as normalized against renilla luciferase activity, and representative of six independent experiments, each performed in triplicate. The amount of transfected plasmid DNA was maintained constant by adding empty vector. *p<0.05. Bottom right: The same experimental procedures were repeated on Daoy cells using a Dll1 3’-UTR construct with mutations within the miR-34a binding site as the reporter. **B.** Representative Western blot time-courses for Daoy (B) and D283-MED (D) cells transfected with miR-34a, using a panel of antibodies against: Dll1, NICD1, Hey1, NICD2, Hes1 and β-Actin. **C.** Luciferase assay on Daoy cells co-transfected with the CBF1/RBPj-k reporter construct, the pGL3 control vector, and the wild-type or seed-mutated miR-34a. Luciferase activity at 14 h from transfection, was normalized against renilla luciferase activity. Data are representative of six independent experiments, each carried out in triplicate. The amount of transfected plasmid DNA was maintained constant by adding empty vector. *:p<0.05 **D.** Representative Western blot for Daoy cells 10 h from transfection with wild-type or seed-mutated-miR-34a, or at 72 h from treatment with SNALPs carrying miR-34a or SNALP-scrambled, using an anti-Dll1 antibody. Non-transfected Daoy cells were used as control. **E.** Representative Western blot as for (B) on D283-MED cells transfected with miR-34a **F.** Representative Western blot as for (B) for stable miR-34a clones 1 and 2, a stable empty vector clone, and wild-type Daoy cells.

Transfection of the miR-34a-expressing vector significantly down-regulated Dll1 reporter activity in Daoy MB cells, while no significant inhibition was seen for the Jag1, Notch1 and Notch2 3’-UTR reporters (Fig. 1A). Of note, mutation of the three seed sequences within the 3’-UTR of Dll1 completely abrogated this suppression effect of miR-34a. Mutation of the miR-34a seed-region from the 2^nd^ to the 4^th^ base (miR-34aMut) also resulted in a lack of binding of this miR-34aMut to the Dll1 3’-UTR region. These results suggest that miR-34a regulates Dll1 expression through three binding sites in the 3’-UTR of the gene that encodes Dll1 (Fig. 1A).

We then asked whether miR-34a can affect the endogenous expression of Dll1. As Dll1 is a known ligand of the Notch1 and Notch2 receptors [21], we investigated whether miR-34a expression can influence the regulation of both of these genes and their pathways.

*In-vitro* studies have already shown that miRNAs can induce translational inhibition in a very short time frame [22]. Therefore, the effects of miR-34a on Notch signaling were investigated in a time-dependent manner, following time-courses in Daoy MB cells from 10 h to 16 h after miR-34a transfection. MiR-34a expression resulted in a transient reduction in Dll1 protein levels by 10 h (Fig. 1B). At this time, no decrease in Dll1 mRNA levels was detected (data not shown), suggesting an initial effect of miR-34a on Dll1 translation, and then later on Dll1 mRNA cleavage. On the other hand, the recovery of the Dll1 protein levels at 12 h (Fig. 1B) was also supported by a transitory increase in its mRNA levels (data not shown), which might have been due to inherent positive-feedback-loop mechanisms between Notch1 and Dll1 already described [23], [24].

Dll1 down-regulation was followed by rapid activation of Notch1, as shown by the detection of the Notch1 intracellular domain (NICD1) protein at 12 h (Fig. 1B). The activation of Notch1 downstream signaling was confirmed by HEY1 protein expression (Fig. 1B) and also by induction of CSL1 transcription factor reporter activity, which was detected at 14 h from miR-34a transfection (Fig. 1C).

MiR-34a overexpression also resulted in transient inhibition of Notch2 signaling 12 h post-transfection, as seen by down-regulation of NICD2 and of its known target: the Hairy and enhancer of split 1 (Hes1) proteins (Fig. 1B). Of note, Notch1 activation and Notch2 inhibition are not likely to be the result of gene expression modifications, as the mRNA levels did not follow the same trends in their expression (data not shown). To further validate the previous findings, expression of miR-34a was also determined at each time used for the protein expression analyses (Fig. S1A).

Consistent with the luciferase assay data, miR-34aMut transfection did not have any effects on Dll1 protein levels (Fig. 1D). This confirmed the transient specific down-regulation operated by miR-34a on Dll1.

Time-course experiments showed similar results in two other cell lines derived from classic MB tumor types. In both D283-MED (Fig. 1E) and UW-228 cells (Fig. S1B), there was down-regulation of the Dll1 protein at 10 h post-transfection, which was then followed by strong Notch1 activation. At the same time, Notch2 signaling was inhibited, and the Hes1 protein was down-regulated (Fig. 1B).

Altogether, these data indicate that the ectopic expression of miR-34a in MB cells can transiently down-regulate Dll1 protein levels, and also influence Notch1 and Notch2 signaling.

We further investigated the roles of Notch1 and Notch2 in MB tumor biology in which their opposite effects have already been reported: Notch1 activity inhibits cell growth and induces apoptosis, while Notch2 up-regulates Hes1 expression, which promotes cell proliferation [25]. To dissect out these functions, we generated two different miR-34a-expressing stable clones in Daoy MB cells (Fig. 1F), and then we analyzed this pathway, using Western blotting.

Here, we observed that the two miR-34a-expressing clones showed sustained reductions in Dll1 protein levels and marked down-regulation of NICD2 and Hes1 protein expression (Fig. 1F). In these two clones, we also noted only in clone#1 a weak activation of Notch1 (Fig. 1F). This activation can be explained by the relatively high expression of miR-34 in this clone, as compared to clone #2 (Fig. S1C). To further validate the direct down-regulation of the expression of the Dll1 protein by miR-34a, we used a miR-34aMut and Stable Nucleic-Acid-Lipid Particles (SNALPs) carrying mature miR-34a. While both the miR-34a wild-type precursor and the SNALPs carrying the mature miR-34a down-regulated Dll1 expression, miR-34aMut and SNALP-scrambled (SNALPs containing an unrelated oligonucleotide) did not function, further indicating that there is direct functional regulation by miR-34a on Dll1 protein expression (Fig. 1D).

**Figure 2:**
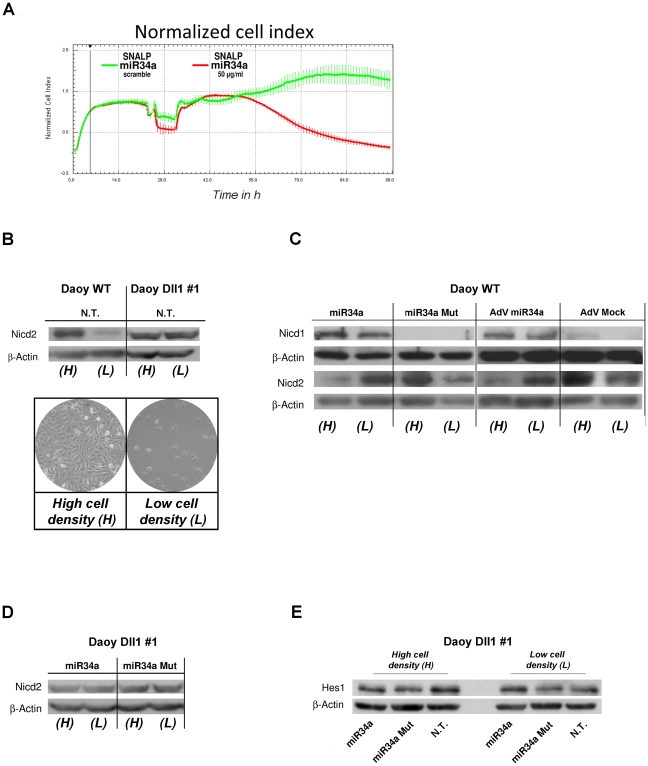
Opposite effects of MiR-34a on Notch1 and Notch2 receptors throw the direct targeting of Dll1 in the MB Daoy cell line. **A.** Normalized cell index (means ±SD) as a measure for proliferation of Daoy cells treated with SNALPs carrying miR-34a or with SNALP-scrambled. Treatment was initiated 20 h post seeding. **B.** Representative Western blot for Daoy WT and Daoy stable Dll1 clone #1. The cells were plated at high density (H) or low density (L), as illustrated. Anti-NICD2 and anti-β-Actin antibodies were used. **C.** Representative Western blot for Daoy cells plated at different densities and transfected with wild-type or seed-mutated miR-34a, and infected with adenovirus carring AdV-GFP-miR-34a and AdV-GFP-mock using anti-NICD1, anti-NICD2 and anti-β-Actin antibodies. **D.** Representative Western blot for Daoy stable Dll1 clone #1 cells plated at different densities and transfected with wild-type or seed-mutated miR-34a, using, anti-NICD2 and anti-β-Actin antibodies. **E.** Representative Western blot analysis for Daoy Dll1 clone #1 cells plated at different densities, under basal conditions or at 14 h from transfection with miR-34a or with an empty vector, using anti-Hes1 and anti-β-Actin antibodies.

Previous studies have demonstrated that the soluble dominant-negative form of Dll1 inhibits cell proliferation in Daoy and D283-MED MB cells [26]. We thus asked whether by targeting Dll1, miR-34 can impair the proliferation rate of MB cells. Measuring proliferation according to the 3-(4,5-dimethylthiazol-2-yl)-5-(3-carboxymethoxyphenyl)-2-(4-sulfophenyl)-2Htetrazolium (MTS) cell-proliferation assay (see Supporting Information S1, Materials and methods), there was a statistically decrease in the proliferation rates between the Daoy miR-34a stable clones and the control parental cell line (Fig. S1D). MiR-34a transient transfection impaired the proliferation of MB ONS-76 and D283-MED cells (Fig. S1F). Using cell index assays, we confirmed that with the SNALP carrying miR-34a, these Daoy MB cells show impaired proliferation (Fig. 2A). These data are of importance for demonstration of the potential therapeutic use of this technology *in vivo*. We also investigated whether miR-34a was able to influences similarly both Notch 1 and Notch2 receptor signaling in MB cells through its down-regulation of Dll1, considering both cell autonomous (ligand and receptor expressed within the same cell) and non-autonomous [27], [28] (ligand and receptor expressed by two distinct, but neighboring, cells). To achieve this, we generated a Dll1-expressing stable clone in the Daoy MB cells (Daoy-Dll1#1) (see Fig. S1G) and we performed Western blotting on these Daoy and Daoy-Dll1#1 cells, taking into account the cell-cell contact. We used a “high-cell-density context (H)” (3×10^5^ cells/cm^2^ at seeding), where the cells were in contact each other, which would demonstrate cell non-autonomous Notch signaling, and a “low-cell-density context (L)” (0.5×10^5^ cells/cm^2^ at seeding), where cell-cell contact was not evident, which would demonstrate cell autonomous Notch signaling (Fig. 2B). At steady-state at the high cell density, Dll1 expression was 5-fold more elevated than that of Notch1, and 5-fold less than that of Notch2 (Fig. S1E). At the same time, up-regulation of NICD2 demonstrated that Notch2 was activated only at the high cell density in the Daoy cells, while in the Daoy Dll1-expressing stable clones (Daoy-Dll1#1), Notch2 was activated at both the high and low cell densities (Fig. 2B); the NICD1 protein was not detectable under these conditions (data not shown). Thus, transient overexpression of MiR-34a, using both transfection and adenovirus infection, inhibits Notch 2 activation, which reduces the levels of the NICD2 protein in the high-cell-density context, but which does not occur at the low cell density (Fig. 2C) further suggesting the existence of additional mechanisms controlled by miR34a that might be due an activation of Notch2 “cell autonomous signaling”. Further studies should properly address these findings in the near future. Conversely, while expression of miR34a inhibited Notch2 at high cell density impairing this signaling, this phenomena did not have similar effects on Notch1 (analyzed by measuring the amounts of activated NICD1 protein). When this analysis was performed on Notch 1 signaling, we show that NICD1 was activated both at high and at low cell density (Fig. 2C). This thus indicates that in Daoy MB cells, Dll1 act as a repressor on Notch1, and also that through its direct down-regulation effect, miR-34a can then activate Notch1 signaling.

Moreover, in this Daoy-Dll1#1 clone, we observed that miR-34a does not negatively influence Notch2 activation (Fig. 2D), both for the high and low cell density context; these results are further supported by no variations in the Hes1 protein levels (see Fig. 2E).

Altogether, these results demonstrate that in MB, miR-34a overexpression controls both *autonomous* and *non autonomous* Notch signaling through direct down-regulation of the Dll1 target.

### MiR-34a action within a gene-target network

**Figure 3:**
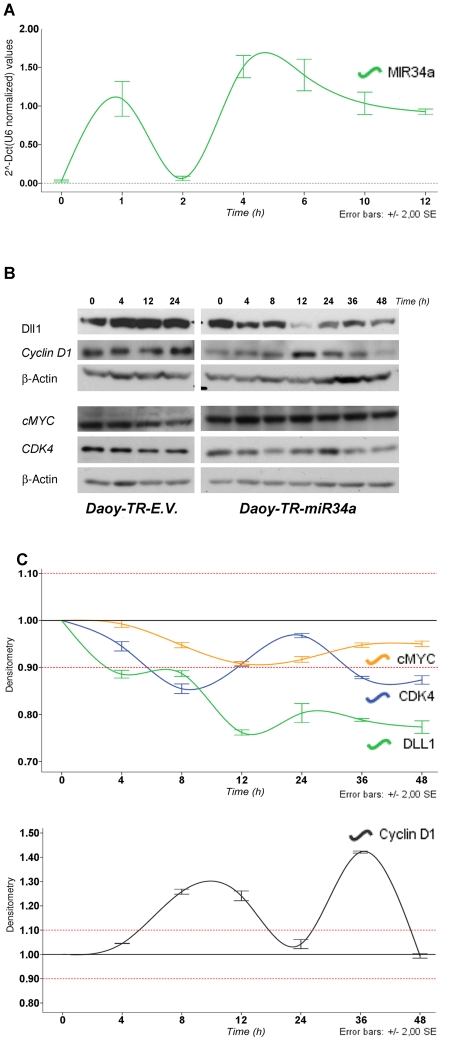
MiR-34a tetracycline inducible on-off model: gene-target network. **A.** Real-time PCR showed the time-dependent expression of miR-34a following tetracycline single-pulse stimulation. Data are means ±standard deviation of 3 experiments, each carried out in triplicate. **B.** Top: Representative Western blot for time-course of tetracycline-stimulated Daoy-TR-EV and Daoy-TR-miR-34a cells, using an antibody panel against: Dll1, CyclinD1, cMYC, CDK4 and β-Actin. **C.** Densiometric time-course analyses (Dll1, cMYC, CDK4), as β-Actin normalized, each value was expressed as fold-stimulation over the unstimulated cells (t0). Data are means ±standard deviation of 3 experiments, each carried out in triplicate.

One debatable question raised at this time relates to miR-34a target recognition, following the identification of several gene targets for miR-34a. Understanding the gene-target network of miR-34a will be of importance for future therapeutic applications. For this reason, we sought to verify if some of reported targets in the literature (e.g., Cyclin D1, cMyc, CDK4) are down-regulated together with Dll1 by miR-34a in a time-dependent manner in our cell model. We choose those targets because they are all affecting several concerning pathways within cell cycle involved into proliferation processes of MB cells. To achieve this, we generated several Daoy-TR–miR-34a tetracycline-inducible clones, one of which was here characterized (Daoy-TR-miR-34a) (See Fig. S2A). This clone was further characterize on its capabilities to activate NICD1, by observing that overexpression of mir34a by tetracycline, result on an up-regulation of NICD1 at 8 hours of induction (see Fig. S2B). Through this technology, we evaluated (at different time points) the levels of the miR-34a protein targets following tetracycline induction, comparing both the non-stimulated and the control Daoy-empty vector tetracycline-inducible cell line (Daoy-TR-EV). This system was important to minimize the variability of miR expression during transient transfection and to minimize the side effects on RISC complex obstruction, phenomena that are often encountered once a given miRNA is constitutively expressed. Here, in these Daoy-TR-miR-34a cells, we observed that single-pulse tetracycline stimulation promoted an enhancement of miR-34a expression, as a pulse of expression at 1 h after stimulation, followed by a rapid down-regulation at 2 h, and then again an enhancement of miR-34a expression at 4 h, followed by a decrease of expression to 12 h (see Fig. 3A). In these clones, miR-34a induction lead to early down-regulation of Dll1 at 4 h, followed by an massive down-regulation of the Dll1 protein levels at 12 h. Within this assay, we found that Cyclin D1 was down-regulated at 48 h after induction. C-Myc was not down-regulated by miR-34a induction, neither at the early or the late time points. Cdk4 was also down-regulated at early time points (at 8 h), later than with Dll1(see Fig. 3B). Figure 3C gives a graphic representation of the levels of down-regulation of targets by expression of miR-34a upon tetracycline induction. To investigate whether miR-34a enhancement in these Daoy-TR-miR34a influences also the expression of Cdk inhibitors (p21 and p27 proteins), we performed time course experiment upon single-pulse tetracycline stimulation. Real time experiment showed that in Daoy-TR-miR34a both p21 and p27 mRNA were found upregulated from 2 h to 12 h following miR34a expression. 24 h later tetracycline stimulation, when the miR-34a upregulation was exhausted, p21 and p27 expressions were found downregulated.

Moreover to evaluate whether this observed phenomena wasn’t due to a tetracycline side-effect we performed the same experiment in Daoy-TR-E.V. clone, observing no appreciable variations of p21 and p27 mRNA expression. Then we had validated by WB analysis p21 (at 6 h upon tetracycline stimulation), confirming the result observed previously through realtime mRNA expression analyses.

We additionally explored whether or not the protein half-life during its degradation by the proteasome regulates this observed phenomena of miR-34a controlling Dll1 expression. If miR-34a attenuated the accumulation of Dll1 induced by the MG132 proteasome inhibitor, we could gain additional indirect evidence that miR-34a down-regulates Dll1. For this purpose, we monitored the levels of the Dll1 protein following time-course Western blotting using Daoy-TR-miR-34a cells. Dll1 started to accumulate at 6 h from MG132 administration. As expected, in the presence of both MG132 and tetracycline, the Dll1 protein was not degraded, although due to the miR-34a induction, it was not accumulated either (see Fig. S2B). To exclude that the maintained presence of the Dll1 protein was due to the presence of miR-34a and not to the tetracycline, this was repeated with Daoy-TR-EV control cells, which do not overexpress miR-34a in response to tetracycline. As expected, tetracycline did not influence the accumulation of Dll1 induced by MG132, as illustrated in Figure S1B. These data further demonstrate that Dll1 is one of the first targets regulated in MB (in comparison with CyclinD1, cMyc, CDK4), and also that miR-34a affects the Notch pathway, driving additional signals that will be further investigated.

### Negative targeting of Dll1 by MiR-34a influences apoptosis

**Figure 4:**
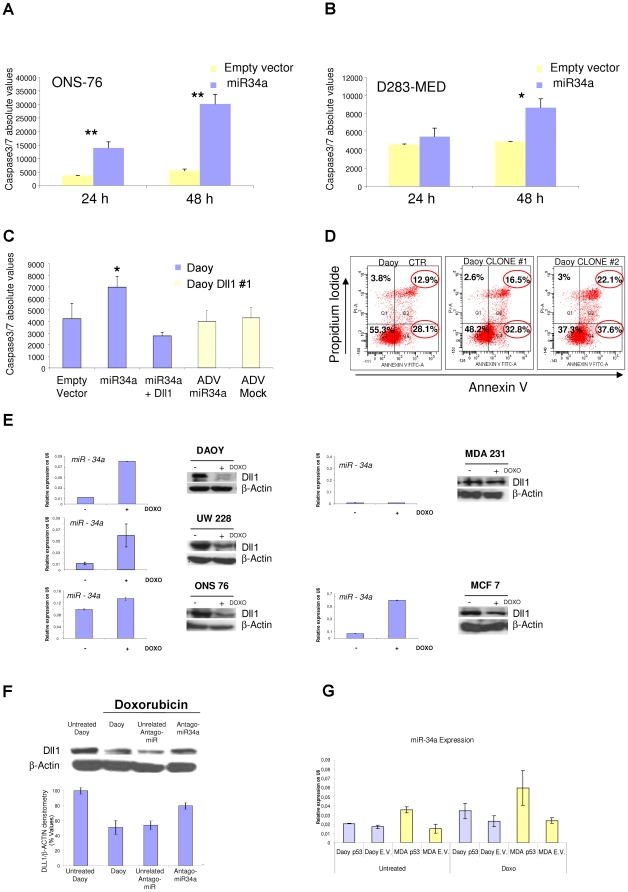
Apoptosis analysis of MB cells upon miR-34a expression, and doxorubicin stimulation of MB and breast cell lines. **A, B.** Caspase 3/7 assays carried out in ONS-76 (A) and D283-MED (B) cells, at 24 h and 48 h after transfection with miR-34a or empty vector. **C.** Caspase 3/7 assay performed in Daoy cells 24 h from co-transfection with miR-34a and the empty vector or with miR-34a and the mouse Dll1-expressing vector; and in a Daoy Dll1 stable clone, at 48 h after infection with AdV-GFP-miR-34a or AdV-GFP-mock viruses. (A-C) Data are means ±standard deviation of 3 experiments, each carried out in triplicate. *:p<0.05, **:p<0.005 **D.** FACS analysis for basal apoptosis of Daoy miR-34a stable clones (clones 1 and 2) and of a Daoy empty-vector stable clone, grown under the same selection conditions. Percentages of cells in early and late apoptosis (Q2 and Q4 squares, respectively) are marked with red circles. **E.** Real-time PCR showing miR-34a expression and representative Western blots showing Dll1 expression of MB (Daoy, UW228 and ONS76) and breast (MCF7 and MDA231T) cells lines, upon 24 h of doxorubicin stimulation. Untreated cells were used as control. The real-time PCR reactions were normalized to mU6. Data are means ±standard deviation of 3 experiments, each carried out in triplicate.*p<0.05. **F.** Representative Western blot of Daoy cells, using anti-Dll1 and anti-β-Actin antibodies. At 12 h from transfection with miR-34a antago-mir or with an unrelated antago-mir, the cells were treated with doxorubicin for 12 h. Untreated cells, or treated and non-transfected Daoy cells, were used as controls. Densitometric quantification of Dll1 protein is reported below, as means ±standard deviation of 3 different experiments. **G.** Real-time PCR showing miR-34a expression in Daoy, and MDA-231T cells lines transfected with p53 wt, and treated for 12 h with doxorubicin, 18 h later transfection. Empty vector trasfected cells were used as control. The real-time PCR reactions were normalized to mU6.

To further confirm that miR-34a has a central role in apoptosis, we evaluated its effects on caspase activation. Following ectopic expression of miR-34a, there was substantial activation of apoptosis in the MB ONS-76, D283-MED and Daoy cell lines (Fig. 4A-C), which resulted from massive caspase activation (as activation of caspases 3/7; p≤0.002; p≤0.02 and p≤0.02, according to cell types, respectively).

Altogether, these data indicate that in MB cells, miR-34a impairs proliferation *in vitro*, which induces apoptosis. ‘Rescue’ experiments using Daoy cells that were stable for the Dll1 cDNA that lacked the 3’-UTR that contained the miR-34a binding sites, attenuated the miR-34a pro-apoptotic effects (Fig. 4C) (measured by caspases 3/7 activity), thus suggesting that in the Daoy cells, direct down-regulation of Dll1 miR-34a is involved in caspase-driven apoptosis.

These data are in agreement with those reported by Raver-Shapira et al. (2007) [29] in U2OS human osteosarcoma cells. This hypothesis was further confirmed by fluorescence-activated cell sorting (FACS) analyses, using annexin V and propidium iodide staining in the above-described miR-34a stable clones. As shown in Figure 4D, miR-34a-expressing clones had a higher fraction of apoptotic cells, compared to the empty vector control clone [30]. Moreover, *in-vitro* tumorigenicity assays also showed significant reductions in the soft-agar colony formation in both of the cell lines analyzed here (Fig. S3A, B).

Taken altogether, these findings suggest that miR-34a expression has a pro-apoptotic effect and impairs soft-agar colony formation in MB cells.

### MiR-34a endogenous expression and regulation by p53 activation

To investigate further the functional effects of endogenous miR-34a expression in MB cells, we stimulated UW228, ONS76 and Daoy cells with the genotoxic agent doxorubicin [11], a known p53 inducer. Doxorubicin can potentiate miR-34a transcriptional activation, as already shown by other investigators in other cell lines [31] and as here verified in MB cells (Fig. 4E). As the control, we used the mRNA levels of the known p53 downstream gene *P21-WAF1* (Fig. S3C). Although Daoy cells harbor a p53 homozygote mutation (C242F), they retain a 22,8% p53 promoter-specific transcriptional activity, as measured in yeast functional assays on WAF1 (expressed as percent of wild-type activity; see additionally the data from a p53 database available at “http://www-p53.iarc.fr/”), because of these reported data we reason doxorubicin stimulation may be due in Daoy cells to an enhancement of miR34a transcription. As observed in other cell lines, our data show that endogenous miR-34a up-regulation by doxorubicin negatively influences the expression of Dll1, and this confirms our previous data using transient miR-34a regulation. Then, we asked whether this process can be generalized to other tumor cell types. For this purpose, we used the MCF-7 and MDA-231-T human breast cancer cell lines, which have, wild-type and mutated p53 forms, respectively, as previously reported [32]. Doxorubicin stimulation caused miR-34a induction in ONS-76, UW-228, Daoy and MCF-7 cells (Fig. 4E). As expected, miR-34a expression was not induced in the treated MDA-231T breast cancer cells, which have R280K p53 mutation, that led to an p53 transcriptional activity measured as 0,8%, making these cells unresponsive to doxorubicine treatment [33]. This provides further evidence of direct regulation of endogenous miR-34a on Dll1 expression once it is activated by p53.

We saw here additionally that doxorubicin stimulation resulted in Dll1 protein down-regulation (Fig. 4E), despite the increase in Dll1 mRNA levels detected in doxorubicin-treated cells (Fig. S3D, E). Remarkably, transfection of the miR-34a-2’-O-methyl antisense oligoribonucleotide (miR-34a-2-O-Me) partially recovered the Dll1 protein levels (Fig. 4F). Altogether, these data indicate that the endogenous levels of miR-34a can regulate Dll1 protein expression. Moreover we had performed additional treatments to verify wheter restoration of p53 wild tipe (wt) isoform in both Daoy and MDA-231-T cell lines led to an enhancement of miR-34a expression. Those cells were transfected with p53 wt and 18 h later were stimulates with doxorubicin for 12 h. Real Time experiment was performed, to evaluate miR34a expression, using p21 expression as control. In this experiment, we shown that in Daoy cells, transfected with p53 wt expressing vector, doxorubic treatment enhance further mir34a expression compared to Daoy cells transfected with empty vector. On the other hand in MDA-MB-231 cells, which were unresponsive to doxorubicine treatment, wt-p53 transfection led to an increase of miR34a and p21 expression, both in untreated and doxorubicin treated cells (see Fig. 4G, and Figure S3F).

### MiR-34a influences inhibition of MB tumor-propagating cells, inducing neural differentiation

Tumor growth depends on a subset of tumor cells that are known as TPCs. To investigate the role of miR-34a on the proportion of TPCs, we infected human MB Daoy cells for 12 h under 20% and 1% oxygen conditions (normoxia and hypoxia, respectively), with an adenovirus-type-V-containing miR-34a precursor, followed by an IRES-driven green florescent protein (GFP) vector.

**Figure 5:**
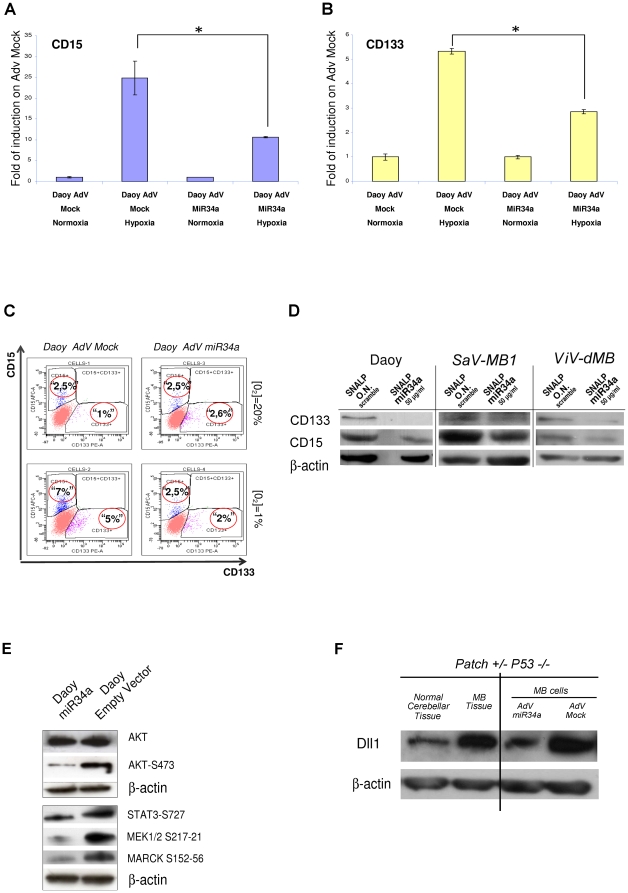
Decrease in CD15^+^ and CD133^+^ expression in Daoy cells under hypoxia condition, upon miR-34a overexpression. **A, B.** Real-time PCR showing CD15 (A) and CD133 (B) expression in Daoy cells grown under normoxia and hypoxia conditions (as indicated) for 12 h, after 12 h of infection with AdV-miR-34a or AdV-GFP-mock viruses. Fold-changes are shown with respect to CD15 and CD133 expression, as measured in AdV-GFP-mock infected cells. Data are means ±standard deviations of 3 experiments, each carried out in triplicate *: *p*<0.05. Real-time PCR reactions were normalized to β-Actin. **C.** Representative FACS analysis for CD15^+^ and CD133^+^ subpopulations in Daoy cells grown under normoxia or hypoxia conditions for 12 h, after 24 h of infection with AdV-GFP-miR-34a or AdV-GFP-mock. **D.** Representative Western blot for Daoy cells and two primary human MB cell lines (SaV-MB1 and ViV-dMB) at 72 h of treatment with a SNALP carrying miR-34a or with a SNALP-scrambled, performed using anti-CD133, anti-CD15 and anti-β-Actin antibodies. **E.** Representative Western blot with a Daoy miR-34a stable clone and a Daoy empty-vector stable clone, using an antibodies panel against: Ak, Akt-S473, STAT3-S727, MEK1/2 S217-221, MARCK S152-156 and β-Actin. **F.** Representative Western blot of normal mouse cerebellum, and Patch^+/-^ P53^-/-^ and primary Patch^+/-^ P53^-/-^ mouse MB cell lines, at 48 h from infection with AdV-GFP-miR-34a or AdV-GFP-mock viruses, carried out using anti-Dll1 and anti-β-Actin antibodies.

We obtained maximum efficiency of infection of those cells, and determined the levels of endogenous CD15 and CD133 mRNAs, under these normoxia and hypoxia conditions (Fig. 5A, B; Fig. S3F). As revealed using quantitative real-time-PCR, there were significant reductions in both CD15^+^ and CD133^+^ expression in the Daoy AdV-GFP-miR-34a infected cells (p<0.05, p<0.01, respectively) (Fig. 5A, B), as compared to the AdV-GFP-mock-infected cells, and this effect was enhanced in the cells subjected to hypoxia. Thus, in these Daoy cells, miR-34a overexpression reduced the proportion of TPCs from 7.0% to 2.5% and from 5.0% to 2.0%, respectively (Fig. 5C). These results were further validated by Western blotting in Daoy cells and in two primary cell cultures extracted from human MBs (classic and desmoplastic) using SNALP-containing oligonucleotides for both miR-34a and an unrelated scrambled oligonucleotide sequence (Fig. 5D). Further evidence of inhibition of the proportion of TPCs came from immuno-fluorescence analyses using NESTIN (a marker of neuronal precursor cells [NPCs]) and glial fibrillary acidic protein (GFAP; a specific glial neuronal cell marker) immuno-staining, as shown in Fig. S4A. These data indicate a reduction in NESTIN staining in these miR-34a-AdV infected Daoy cells, as compared to the AdV-mock-infected cells, then we saw a strong staining with GFAP seen in the AdV-miR-34 cells, thus showing clear signs of differentiation.

We also wanted to understand which other intracellular signaling pathways are affected by miR-34a deregulation. To achieve this, we used reverse-phase proteomic arrays [34]. We analyzed cell lysates from four independent Daoy miR-34a stable clones, and compared the data obtained with those from Daoy empty-vector stable clones. We observed that in these miR-34a overexpressing clones, the proportion of the active Akt kinase protein (Akt S473) was decreased, while the Akt protein levels did not vary, as validated by Western blotting (Fig. 5E, S4B). We also found PTEN phosphorylated on T380 (Fig. S4B), a sign that pro-apoptosis signaling was occurring in these miR-34a overexpressing clones. Finally, the phosphorylation of S727 of STAT-3 was down-regulated in these miR-34a overexpressing clones (Fig. 5E, S4B). Additional putative targets are discussed in Supporting Information S1 Material section. Altogether, these data support our previous findings and correlate miR-34a function with inhibition of Akt/phosphoinositide 3-kinase (PI3K)/PTEN signaling, which is responsible for maintenance and propagation of TPCs.

An additional question was raised whether other miR-34 family members can have synergistic actions on Dll1 down-regulation. For this reason, we performed additional Dll1 3’-UTR reporter activity assays using miR-34b- and miR-34c-containing expression constructs, and showed that both miR-34b and miR-34c down-regulate Dll1 3’-UTR to the same levels as those seen with miR-34a (Fig. S5D). These data provide further supporting evidence that the whole miR-34 family (miR-34a, miR-34b and miR-34c) can regulate Notch signaling through Dll1 in MB.

### P53–MiR-34a–Dll1 axis and functional differentiation assays

Several studies have reported a requirement for Dll1 for maintenance of undifferentiated NPCs. In central nervous system development, Dll1 is the major ligand for the Notch receptor and it contributes to maintenance of the undifferentiated state of NPCs [35]. Moreover, transgenic Dll1^LacZ^ mice show high Dll1 activity in the cerebellum, and in particular, in the Purkinje cell populations at the margins of the molecular and granular cell layers within the cerebellum [36]. In MB, miR-34a Daoy stable clones in which we found Dll1 constitutively down-regulated showed a differentiated phenotype, with an increased level of the glial fibrillary acidic protein (GFAP), as assessed by real-time PCR (Fig. S4C) and by morphological and immunofluorescence analyses (Fig. S4D). Here we sought to investigate the potential therapeutic function of miR-34a in a mouse model of MB.

We evaluated first the Dll1 protein levels in the murine MB model of Patch^+/-^ p53^-/-^ mice (the most representative animal model of MB) (Fig 5F). There was a substantial increase in Dll1 protein levels in the tumor compared to the healthy cerebellum. Since miR-34a precursor sequence is evolutionarily conserved, as is the Dll1 3’-UTR sequence, we determine whether human miR-34a can also regulate murine Dll1 in Patch^+/-^ p53^-/-^ MB mouse model (Fig 5F). Tumorigenic cells were isolated from Patch 1^+/-^ p53^-/-^ mice and were infected with AdV-GFP-miR-34a and AdV-GFP-mock 48 h later, Western blotting was carried out, which demonstrated that human miR-34a impaired mDll1 protein expression in the Patch mouse MB cells. These results prompted us to investigation this animal model further. For this reason, we isolated tumor spheres [19] from both Patch 1^+/-^ p53^-/-^ and Patch 1^+/-^ p53^+/-^ mice, and used miR-34a to look for any effects on cell differentiation.

**Figure 6:**
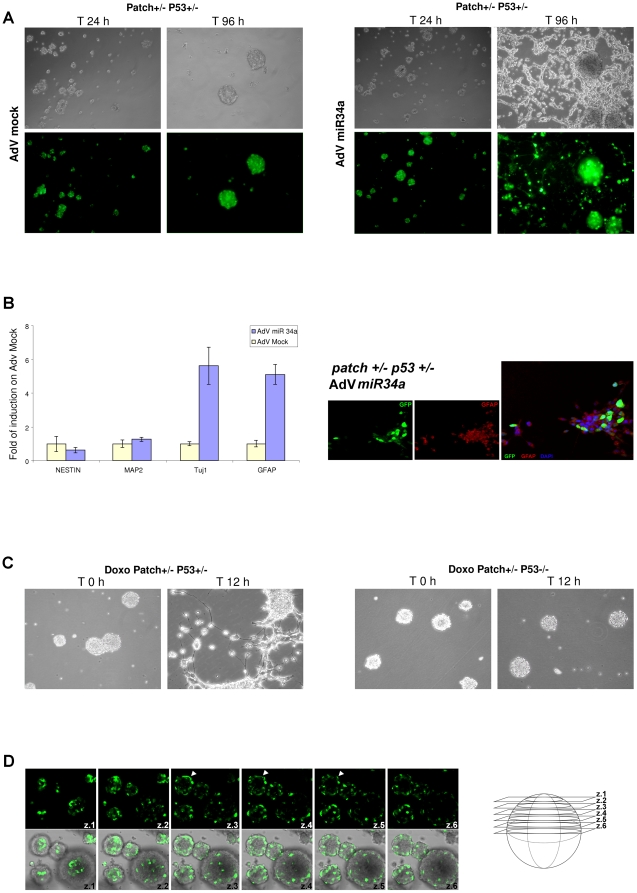
Neural differentiation of tumor spheres by miR-34a. **A.** Differentiating effects of AdV-GFP-miR-34a on tumor spheres from Patch^+/-^ P53^+/-^ mice. Representative microscopy images and confocal GFP immunofluorescence staining of Patch^+/-^ P53^+/-^ mouse MB spheres at 24 h and 96 h from AdV-GFP-mock (left) or AdV-GFP-miR-34a (right) virus infections. **B.** Left: Real-time PCR analysis showing expression levels of the neural markers Nestin, MAP2, TUJ1, and GFAP in Patch^+/-^ P53^+/-^ tumor spheres at 96 h from infection with AdV-miR-34a or AdV-mock viruses. Fold changes are shown, calculated with respect to the gene expression of the AdV-mock infected tumor-spheres. Data are means ±standard deviations of 3 experiments, each carried out in triplicate. Real-time PCR reactions were normalized to β-Actin. Right: Representative immunofluorescence staining of Patch^+/-^ P53^+/-^ tumor spheres, differentiated following viral delivery of miR-34a, performed using an anti-GFAP antibody. GFP indicates the infection efficiency and the tumor sphere viability. **C.** Doxorubicin treatment of Patch^+/-^ P53^+/-^ and Patch^+/-^ P53^-/-^ tumor spheres. Representative microscopy images showing the neural differentiating phenotype observed only for the P53^+/-^ tumor spheres. **D.** Left: Representative confocal GFP immunofluorescence staining of Patch^+/-^ P53^+/-^ tumor spheres at 24 h from AdV miR-34a virus infection. Arroweds denotes that the AdV-miR-34a virus efficiently infects only the cells located in the most external regions of the tumor spheres. Right: Illustration of the cell z-slices imaged.

These tumor spheres changed their morphology 96 h post-infection with AdV-GFP-miR-34a: they differentiated, inducing neurite sprouting (Fig. 6A). Furthermore, real-time PCR analysis showed some 5.5-fold enhancement of TUj1 and GFAP protein expression, as neural and glial cell markers, respectively, in these MB spheres infected with AdV-GFP-miR-34a from both the Patch1^+/-^ p53^+/-^ mice and the Patch1^+/-^ p53^-/-^ mice (Figs. 6B, S6A, S6B and Movie S1). This phenomenon was not observed with AdV-GFP-mock infection of these MB spheres. Additional data show that doxorubicin treatment of these MB spheres from Patch1^+/-^ p53^+/-^ mice induced neural differentiation, while enhancing miR-34a through p53 activation (Fig.6D). We further confirmed these data using an additional p53-positive regulator, Nutlin3 (data not shown). In control experiments, using doxorubicin on these MB spheres from Patch1^+/-^ p53^-/-^ mice, the differentiation phenomena was not observed (Fig. 6C). Those result can be explained by the presence of a functional allele of p53 that can, in turn, induce miR-34a and down-regulate Dll1; this was not seen in cells from the null p53 (p53^-/-^) mice. Additional immunofluorescence analyses of these tumor spheres confirmed our previous findings, showing that miR-34a overcomes the loss of p53 and induces mainly neuronal and glial differentiation (Fig. S6A-C). In Figure 6D, it can be seen that AdV-GFP-miR-34a infection is mainly driven by adenoviruses reaching those cells that are positioned externally in these spheres (see Movie S1) within the tumor-sphere aggregates (Fig. 6D, see arrows on z3-z4 axes), thus showing the potency for miR-34a up-regulation only in these external cells, with the driving of the differentiation processes into the inner neighboring cells. Then, using miR-34a expression, neural differentiation is observed only when the tumor spheres are plated at high density (Fig. 6A, S6A), thus underlining that the p53/miR-34a/Dll1 specific axis influences the differentiation processes in a non-autonomous Notch-signaling manner in MB.

### MiR-34a function in tumorigenic assays in nude athymic mice

**Figure 7:**
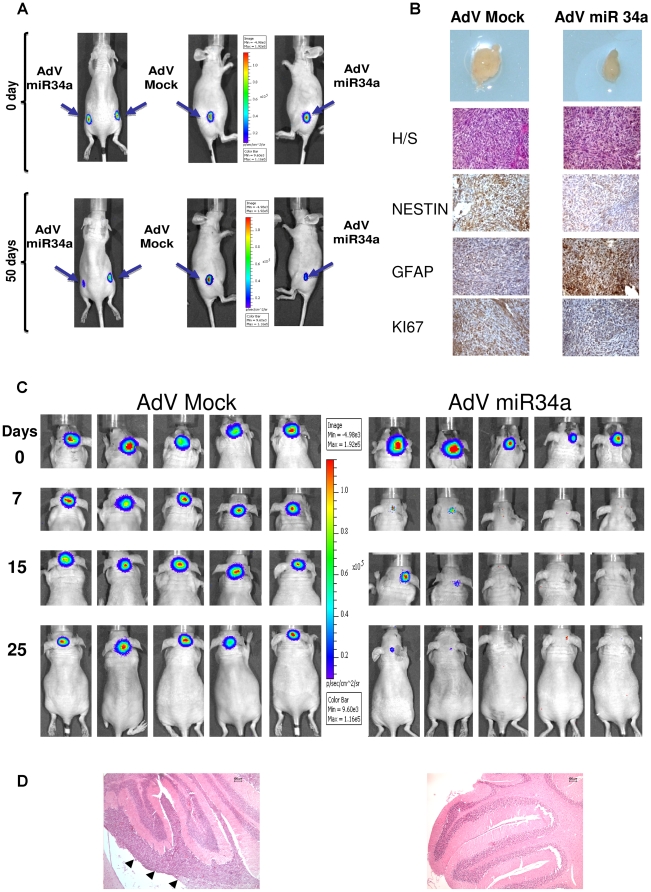
Orthotopic xenografts of MB Daoy cells overexpressing miR-34a by adenovirus infection: functional effects of miR-34a *in vivo*. **A.** BLI of one selected mouse showing development of tumor burden over 50 days. Daoy-Luc cells previously infected with AdV-miR-34a or AdV-GFP-mock viruses were injected into the flanks of the nu/nu mice. **B.** Top to bottom: Tumor size, hematoxylin-eosin and immuno-histochemistry staining of Daoy tumors raised into the flanks of the nu/nu mice, for Nestin, GFAP and KI67. **C.** BLI of five mice injected in the fourth cerebellar ventricle with Daoy-Luc cells previously infected with AdV-miR-34a or AdV-GFP-mock viruses. Photon emission shows that within 25 days there is development and engraftment of the tumor burden with the AdV-GFP-mock that is greater than that with AdV-miR-34a. **D.** Hematoxylin-eosin staining of MB Daoy orthotopic xenografts raised in the nu/nu mice (left) and of a normal cerebellum (right). Arrowheads denotes tumor engrafment. Scale bar 100 µm.

We then sought to investigate these *in-vitro* effects of negative regulation of proliferation, enhancement of caspase activation, reduction in the proportion of TPCs, and induction of neural differentiation in an *in-vivo* tumorigenic assay. Thus, three athymic nu/nu mice received injections in both flanks of luciferase-positive Daoy cells that had previously been infected with AdV-GFP-mock or AdV-GFP-miR-34a; tumor growth was then measured over 50 days by *in-vivo* bioluminescence imaging (BLI). Figure 7A illustrates the negative *in-vivo* regulation of tumorigenesis achieved at 50 days using the AdV-GFP-miR-34a-infected cells. These data thus show significant inhibition of tumor growth *in-vivo* (p< 0.004; Fig. S7A). The histochemistry analyses of the extracted *ex-vivo* tumors, which included miR-34a adenovirus infections, showed inhibition of NESTIN expression and enhanced proportions of the glial-astrocyte neuronal marker GFAP in the tumors (Fig. 7B). There was also inhibition of the Ki-67 marker of cell proliferation, which indicated that there were significant numbers of these cells going through glial–astrocyte differentiation processes, confirming the previous results reported *in vitro* (Fig. S5A, B).

We also investigated whether similar results could be obtained in the cerebellum of the nude mice by following xenograft stereotaxic implantation of tumor cells pre-infected with AdV-GFP-miR-34a. At 25 days post-implantation (Fig. 7C), there was a clear and significant impairment of tumorigenesis in those tumors treated with AdV-GFP-miR-34a, compared with those treated with AdV-GFP-mock (p<0.0057; Fig. S7C, right) as further showed by hematoxylin-eosin staining (Fig. 7D). On the other hand, cerebellum implantation of these Daoy Dll1#1 cell pre-infected with AdV-GFP-miR-34a did not shown impairment of tumorigenesis (Fig. S7B), thus indicating that *in vivo* Dll1 replacement can rescue miR-34a anti-engraftment effects. Similar histochemistry analyses to those used for the mouse-flank model with markers of cell proliferation and differentiation again showed reductions in the proportions of TPCs and an enhancement in the pro-differentiation markers (data not shown).

## Discussion

Here, we have shown that miR-34a targets Notch ligand Dll1 in MB cell lines. In mammals Dll1 has n.3 compared to other putative targets analised, that have at most n.2 potential target sites, predicted several miRNA target prediction tools, and this make of Dll1 the most potential and early targetable mRNA by miR34a. MiR-34a overexpression also results in inhibition of Notch2 signaling and activation of Notch1 in both Daoy and D283-MED MB cells, confirming the inhibitory role of Dll1 on Notch1 activation. This experimentally validated hypothesis is also confirmed *in vivo*, in the ventricular zone of the embryonic mouse telencephalon, whereby expression of Dll1 and activation of Notch1 occur in different cells in a mutually exclusive manner [35].

In our assay miR34a did not target Notch1 and Notch2 as previously presented by Li et al., 2009 in glioblastoma. For this reason we think that Dll1 is the only early target of miR-34a in MB, while Notch1 and Notch 2 expression is then controlled by an unknown secondary level mechainism of regulation upon Dll1 negative regulation. We postulated here an additional function of Dll1 once is repressed by miR34a, translating this effect with a further functional regulation of Notch1 and Notch2 receptors. This regulation is induced at the translational change level of Dll1, and we think is due to differential affinity and binding properties of Dll1 with those receptors proteins. Future studies should address this hypothesis.

MiR-34a overexpression can enhance Notch1 signaling in both autonomous and non-autonomous manners. Indeed, endogenous expression of miR-34a correlates with down-regulation of Dll1 in other, different, tumor types and cell lines, such as for example, breast cancer cells. The tumor suppressor p53 was shown to inhibit Notch processing by transcriptional inhibition of presenilin 1 (PS1) [37]. Our data now suggest a new mechanism by which p53 can interfere with the Notch pathway. Using miR-34 direct up-regulation by doxorubicin, we show here that p53 induction results in the down-regulation of Dll1 via miR-34 transcriptional control.

Ectopic expression of Dll1 rescued miR-34a-mediated apoptosis in Daoy MB cells. At present, the target regulation and involvement of miR-34a expression in a range of additional pathways of MB tumorigenesis have been postulated (such as: Bcl2, E2F3 and N-Myc). Therefore, the oncosuppressor activities of miR-34a are likely to correlate with the down-regulation of more than one target at the same time during tumorigenesis (additional possibilities are: c-Met, cyclin D1, cyclin D6, N-Myc, Sirt1, CREB), and future studies are needed to inter-relate these data with those previously reported for targets of miR-34a. Our first analyses in MB showed that Dll1 is the first target that is down-regulated across a panel of other targets that were analyzed (see Fig. 3B).

We also show here that miR-34a delivery through carrier adenovirus particles can impair tumor growth of Daoy cells, and these data are particularly encouraging, as no signs of toxicity or morbidity were observed in these animals.

In MB tumors, Notch2 and Hes1 overexpression have frequently been observed, and Hes1 correlates with poor prognosis, probably through its transcriptional control role in the maintenance of an undifferentiated state of the cells, and also for its direct control on cell proliferation through transcriptional repression of both p21^CIP1/WAF1^ and p27^KIP1^ [38], [39]. Analysis of miR-34a and its association with other proteins that are involved in Notch signaling will be investigate further in the future. Here, we have demonstrated that miR-34a led to an inhibition of Notch2 activity and a reduction in Hes1 protein levels in MB cells.

We also investigated the use of reverse-phase protein-array technology to determine which other genes/proteins might be influenced by miR-34a in MB. Fan et al. (2009) described a block in the Notch pathway using a specific agent, GSI-18, that depletes CD133^+^ glioblastoma cells and inhibits growth of tumor spheres in xenografts, with decreased Akt and Stat3 phosphorylation status. Down-regulation of Akt phosphorylation on S473 was here observed with miR-34a overexpression in MB cells. CD133^+^/Nestin^+^ cells in both gliomas and MB [40] can survive radiation therapy by activating their Akt pathway [41]. Altogether, our data show a therapeutic benefit on overexpression of miR-34a, as it impairs Akt signaling. Here the results presented *in vitro* by the use of SNALP technology set the basis for their therapeutic uses for the delivering of miR-34a into the cerebellum of affected patients, with this resulting in no signs of toxicity according to the literature data in non-human-primate trials [42].

We present here a model (see Fig. S8) that takes into account the Notch autonomous and non-autonomous cell pathways of activation that can be controlled by p53 activation of miR-34a and inhibition of Dll1 expression. This model should provide a basis for future studies. Within the cell-autonomous context in which miR-34a is up-regulated, an important function arises from the enhancement of both Notch1 and Notch2 signaling, which induces proliferation only in ‘committed’ cells and enhancement of apoptosis derived from the increased number of cells in cycling. Then, in a state of ‘no communication’ between cells (low density of cells (L)), the balance of miR-34a regulation induces preferential Notch1 intracellular signaling activation. Conversely, within the non-autonomous context, miR-34a function down-regulates Notch2 and significantly increases Notch1 signaling, which enhances differentiation of the adjacent, receiving, cells. Only a few cells in which the signal of differentiation passes from one cell to another through contact go into apoptosis. Our model positions miR-34a as the regulator of the Notch–Delta interactions, further supporting the data presented by Sprinzak et al. (2010) [43], where they found that Notch ligand-Delta has two activities: it transactivates Notch function in neighboring cells, and it *cis*-inhibits Notch signaling in its own cell.

At present, there is growing interest in the elucidation of the mechanisms that confer unique properties to tumor propagating cells [44]. Here, through its extensive effects, miR-34a can negatively influence both the CD133^+^ and CD15^+^ populations of both primary MB cell lines and Daoy cells. The data presented here are of great therapeutic value in MB, especially as the enhanced proportion of CD15^+^ cells is predominant in predicting survival with MB [19].

Taken together, our data strongly suggest that miR-34a can be used for future therapeutic and prognostic investigations. Indeed, as an extension of miR-34a target regulation, this aspect should also be investigated in other Notch activated solid tumors.

## Materials and Methods

### Tumor-sphere cells: isolation and culture

MB tumor spheres were generated from Patch^+/-^, p53^+/-^ and Patch^+/-^ p53^-/-^ mice that showed physical and behavioral signs of MB, according to the methods described by [19]. These cells were dissected out and grown in culture using the Weiss Laboratory protocols of the University of California, San Francisco (UCSF, San Francisco, California, USA). They were maintained in Neurobasal-A medium supplemented with 10 U/ml penicillin, 0.1 mg/ml streptomycin, 20 ng/ml basic fibroblast growth factor, 20 ng/ml epidermal growth factor (Sigma Aldrich, Milan, Italy) and 2 mM L-glutamine.

### Adenovirus production

To generate the wild-type miR-34a and the mutant miR-34Mut adenoviruses, the expression cassettes of each construct were cloned into the shuttle vector Ad5 pVQ-K-NpA. The correct sequences were confirmed by automatic DNA sequencing. Virus generation and amplification were performed by ViraQuest (North Liberty, IA, USA). Infection with adenoviruses was performed at a multiplicity of infection (MOI) of 100.

### Mature miRNA SNALPs

The synthetic miRNA oligonucleotides used in this study were obtained from the CEINGE in-house facilities. The mature and scrambled control miRNAs had the following sequences: miR-34a (mirbase#MIMAT0000255): 5’-UgGcAgUgUcUuAgCuGgUuGu-3’, Scramble: GuAaUgUuUgGcUcGuGuGcUg (small capitals letters: 2'-O-CH_3_ substitutions).

The mature miRNAs were encapsulated as single strands in SNALPs using a controlled step-wise dilution method, as described previously [45]. The lipid constituents of the SNALPs were 2, 2-Dilinoleyl-4-(2-dimethyl aminoethyl)-[1], [3]-dioxolane (DLin-KC2-DMA cationic lipid), dipalmitoylphosphatidylcholine (Avanti Polar Lipids), synthetic cholesterol (Sigma) and *N*-[(methoxy poly(ethylene glycol)_2000_)carbamyl]-1,2-dimyristyloxlpropyl-3-amine (PEG-C-DMA), used at the molar ratio of 57.1∶7.1∶34.3∶1.4. Upon formation of the loaded particles, the SNALPs were dialyzed against phosphate-buffered saline and filter sterilized through a 0.2 µm filter before use; these SNALPs were stable as a wet preparation when stored at 4°C for more than 1 month.

### SNALP treatment of the Daoy cell line, and cell proliferation assays

For cell proliferation SNALP treatment, the Daoy cells were trypsinized and seeded into 96-well xCELLigence E-plates (Roche) (8,000 cells/well) according to the manufacturer instructions. The cells were grown in Eagle’s minimum essential medium (Sigma) supplemented with 10% fetal bovine serum, 10 U/ml penicillin and 0.1 mg/ml streptomycin (Celbio Pero, Milan, Italy). After 24 h, the medium was replaced with medium containing 50 µg/mL SNALP miR-34a and its control SNALP-scramble, without fetal bovine serum. After 10 h at 37°C, the medium was replaced with medium containing fetal bovine serum, and the cells were monitored in real-time on the xCELLigence system. Four replicate measurements were obtained per condition.

### Flow cytometry analyses

For the FACS analysis, 500,000 viable cells of the empty vector clone and the miR-34a Daoy stable clones were harvested and stained with propidium iodide and an anti-annexin-V antibody. The cells were analyzed using a FACS Calibur instrument (Becton Dickinson, San Jose, USA). The CD15 and CD133 studies were carried out using the same instrument, with antibodies from Milteny Biotec (Auburn, CA, USA), according to the manufacturer instructions: phycoerythrin-conjugated anti-glycophorin A (CD235a) for CD15, and allophycocyanin (APC)-conjugated antibodies for CD133. In brief, the cells were blocked in Fc receptor blocking reagent, and incubated with the anti-CD15 and anti-CD133 antibodies for 10 min in the dark at 4°C. The cells were then washed and resuspended in phosphate-buffered saline. Cells expressing higher levels of CD15 or CD133 than the immunoglobulin G (IgG) controls were considered positive.

## Supporting Information

Figure S1 **A.** Real-time PCR analysis for miR-34a expression in the Daoy cell line following transfection of miR-34a at each time point from 0 h to 16 h. Real-time PCR reactions were normalized to mU6. Data are means ±standard deviation of 3 independent experiments, each carried out in triplicate. **B.** Representative Western blot time course performed on UW228 cells transfected with miR-34a, using an antibodies panel against: Dll1, NICD1, NICD2, Hes1 and β-actin. **C.** Real-time PCR analysis for miR-34a expression in Daoy miR-34a stable clones. Real-time PCR reactions were normalized to mU6. Data are means ±standard deviation of 3 independent experiments, each carried out in triplicate. **D.** MTS proliferation assay performed on stable Daoy miR-34a clones 1 and 2, on a stable Daoy empty vector clone and on wild-type Daoy cells. **E.** Real-time PCR showing Dll1, Notch1 and Notch2 expression in Daoy cells grown under conditions. Fold changes are shown respect to Dll1 expression. Real Time PCR reaction were normalized to β-Actin. Data are means **±**SD from three independent experiments, each carried out in triplicate. **F**. MTS proliferation assay performed on ONS76 and D283 cell lines, both transfected with a vector carrying miR-34a or with an empty vector. **G.** Representative Western blot showing Dll1 overexpression in Daoy Dll1 stable clones 1, 2, 3,4 and 5, with respect to that of an empty vector stable clone, performed by using anti-Dll1 and anti-β-actin antibodies. **H.** MTS proliferation assay performed on Daoy Dll1 stable clones, infected with AdV-miR-34a or AdV-GFP-mock virus, or under basal conditions. Data are means **±**SD from three independent experiments, each carried out in triplicate.
(TIF)

Figure S2 **A.** Real-time PCR showing miR-34a expression in Daoy–miR-34a tetracycline inducible clones (Daoy-TR-miR-34a) at 4 h from tetracycline stimulation, as normalized to sn-U6. Data are means **±**SD from three independent experiments. **B.** Representative Western blot time courses performed on Daoy-TR-miR-34a cells with tetracycline stimulation, using an antibody panel against: NICD1 and β-actin. **C.** Top: Representative Western blot time courses using 2.5 µM MG132 proteasome inhibitor, performed on Daoy-TR-EV and Daoy-TR-miR-34a cells, as indicated, without and with tetracycline stimulation, using an antibody panel against: Dll1and β-actin. Bottom: Dll1 densiometric representation, as normalized to β-actin. following the tetracycline stimulated, each value was expressed as fold-stimulation over the unstimulated cells (t0). **D.** Real-time PCR time courses showing p21 expression in Daoy-TR-EV and Daoy-TR-miR-34a cells, treated with tetracycline. he real-time PCR reactions were normalized to β-actin. **E.** Representative Western blot on Daoy-TR-miR-34a cells 6h later tetracycline stimulation, using an antibody panel against: p21 and β-actin. **F.** Real-time PCR time courses showing p27 expression in Daoy-TR-EV and Daoy-TR-miR-34a cells, treated with tetracycline. he real-time PCR reactions were normalized to β-actin.
(TIF)

Figure S3 **A.** MiR-34a overexpression impairs soft-agar colony formation of D283-MED and ONS76 cells. Cells that received miR34a are less tumorigenic compared to untrasfected or empty vector transfected cells (p values<0.001). Representative three fields of each plate are reported on Figure S3B (cell untrasfected and empty vector or miR34a transfected) which were then counted and plotted to produce histograms represented in Figure S3B. **B.** Colony numbers for D283-MED and ONS-76 cells (as indicated) calculated from three representative fields of each plate, with three plates per sample for untransfected and empty vector or miR-34a transfected cells (* p<0.001). **C.-D.-E.** Real-time PCR analysis of induction of p21^waf1^ (C) and Dll1 (D, E) gene expression after 12 h of doxorubicin stimulation in MB Daoy and breast MCF7 and MDA cell lines. Data are means ±ranges of representative duplicate experiment, as normalized to β-actin expression. **F.** Real-time PCR showing p21 expression in Daoy, and MDA-231T cells lines transfected with p53 wt, and treated for 12h with doxorubicin, 18h later transfection. Empty vector trasfected cells were used as control. The real-time PCR reactions were normalized to β-actin.
(TIF)

Figure S4 **A.** Representative immunofluorescence analysis of Daoy cells 48 h from infection with AdV-miR-34a or AdV-GFP-mock viruses, stained for Nestin or GFAP. **B.** Reverse phase proteomic array showing proteins that were down-regulated (top) and up-regulated (bottom) in miR-34a stable clones 1 and 2, compared to an empty vector stable clone. **C.** Real-time PCR showing the expression profiles of the neural markers MAP2, MATH3, TUJ1 and GFAP in miR-34a Daoy stable clones 1 and 2 and in an empty vector stable clone. Data are means ±ranges of representative duplicate experiment, as normalized to β-actin. **D.** Representative phase-contrast microscopy images (Leika DMIL, 40×0.22 magnification), showing morphological differences between an empty vector Daoy stable clone (left) and miR-34a Daoy stable clones 1 (middle) and 2 (right). The miR-34a clones show extensive neurite out-growth processes and a more differentiated phenotype.
(TIF)

Figure S5 **A.** FACS analyses showing cell counts for CD15^+^ and CD133^+^ subpopulations in Daoy cells grown under normoxia or hypoxia conditions for 12 h, after 24 h of infection with AdV-miR-34a or AdV-mock viruses. Data are means **±**SD from six independent experiments, each carried out in triplicate **B.** MTS proliferation assay of Daoy cells transfected with a pool of three different shRNA constructs targeting the Dll1 sequence or with an unrelated shRNA. Data are means **±**SD from six independent experiments, each carried out in triplicate. Significant impairment of proliferation was seen at both 72 h (*p<0.05) and 96 h from transfection (*p<0.04). **C.** Representative Western blot performed using anti-Dll1 and anti-β-actin antibodies on Daoy cells at 72 h after transfection with Sh-Dll1 and with an Sh unrelated. **D.** Luciferase assay on Daoy cells co-transfected with Dll1 3’UTR reporter constructs and an empty vector, or with miR-34a or miR-34b, c, or with the seed-mutated miR-34a or miR34b, c. The relative luciferase activities are shown at 24 h from transfection, as normalized to the renilla luciferase activity. Data are means ±SD of six independent experiments, each performed in triplicate. The amount of transfected plasmid DNA was maintained constant by adding empty vector.
(TIF)

Figure S6 **A.** Confocal GFP staining on Patch +/- P53 -/- mouse tumor spheres at both 24 h and 96h from AdV-miR34a or AdV-GFP-Mock viruses infection, showing differentiating effect of AdV- miR34a. **B.** Real Time PCR performed on Patch +/- P53 -/- mouse tumor spheres at 48 h from infection with AdV-miR34a or AdV-GFP-Mock viruses. AdV-miR34a infected tumor spheres overexpress both miR34a and the neural differentiating markers at GFAP and Tubb3, respect to AdV-GFP-Mock infected tumor spheres. Folds of induction on AdV-GFP-Mock are shown. Data were normalized to sn-U6 and to β-actin. **C.** Real time PCR showing expression levels of TUj1 and GFAP in MB spheres Patch 1 ^+^/^-^ P53^+^/^-^ treated or not with doxorubicin for 12 h, as fold-induction over untreated tumor spheres, normalized to β-actin. **D.** Immunofluorescence analysis of Patch 1 ^+^/^-^ P53^+^/^-^ tumor spheres at 48h from infection with AdV-miR34a or AdV-GFP-Mock viruses, stained with anti-TUj1 or anti-GFAP antibodies. **E.** Immunofluorescence analysis of Patch 1 ^+^/^-^ P53^+^/^-^ tumor spheres previously infected with AdV-GFP-Mock, treated with doxorubicin for 12 h and then stained with anti-TUj1 antibody. GFP signal from AdV-GFP-Mock virus proves cell viability in spite of doxorubicin toxicity. **F.** Confocal GFP staining on Patch +/- P53 -/- mouse tumor spheres previously infected with AdV-GFP-Mock and then treated with doxorubicin for 12 h. AdV- miR-34a does not exert any prodifferentiating effect at either 24 h or 96 h from infection.
(TIF)

Figure S7 **A.** BLI analysis of 3 etherotopic xenografts performed with Daoy cells previously infected with AdV-miR-34a or AdV-GFP-mock viruses. BLI measurements were performed at 25 days post-implantation. P values were calculated comparing the BLI values of the AdV-miR-34a with those of the AdV-GFP-mock xenografts. **B.** BLI from three mice injected in the fourth cerebellar ventricle with DaoyY-Dll1 #1 Luc cells after infection with AdV-miR-34a virus. Photon emission measured at 25 days from implantation shows development and engraftment of tumor burden. **C.** BLI analysis of MB orthotopic xenografts of Daoy cells previously infected with AdV-miR-34a or AdV-GFP-mock viruses. The reported BLI signals are folded on that measured at t0 day. Data are mean BLI values of AdV-miR-34a and AdV-GFP-mock xenografts (n = 5 for each).
(TIF)

Figure S8 Model of the action of miR-34a upon p53 expression and regulation in MB. Cancer stem cells escape from the control of their division and go through neoplastic transformation, becoming TPCs. In MB, this process involves Notch signaling. The model takes into account the control of the p53/miR-34a/Dll1 axis with the Notch cell autonomous and cell non-autonomous pathways. We hypothesize that miR-34a increases the asymmetric division of TPCs at the expense of the symmetric self-renewing division. Within the cell autonomous context (right), miR-34a enhances Notch 2 signaling, which induces cell proliferation. Conversely, within the non-autonomous context, miR-34a enhances the pathway of Notch1, but blocks that of Notch2, which inhibits cell proliferation.
(TIF)

Table S1 MiR-34a targets were selected by examining the output of the indicated miRNA databases. Each database relies on different algorithms of target prediction and uses different read-out scales; e.g. PITA algorithm shows ΔΔG energetic values of the predicted miRNA/mRNA binding, so the more negative the value, the stronger the binding between the miRNA and the given site. For the 3’UTRs of Dll1, Notch1 and Jag1, more than one miR-34a-binding site was predicted. *Among the experimentally validated miR-34a targets, the Met and Bcl2 genes were chosen as references for the score values.
(DOC)

Movie S1 Tumor spheres isolated from Patch1^+/-^ P53^-/-^ mice infected with AdV-miR-34a show sign of induction of differentiation only when they are not dissociated before the infection. At this time, the infected cells remain in contact with each other and are subjected to Notch signaling via the cell non-autonomous pathway. A time-series of phase-contrast brightfield images of the medullpspheres were acquired with an A-Plan Ph1 10× objective for a total time of 72 h, using a Zeiss Axiovert 200M microscope equipped with an Okolab WJ CO_2_ Microscope Stage Incubator, for controlling the temperature and the %CO_2_. The time delay between individual images used here was 15 min.
(AVI)

Supporting Information S1 (DOC)
