# Supplementary figures and images for: MiR-34a Targeting of Notch Ligand Delta-Like 1 Impairs CD15+/CD133+ Tumor-Propagating Cells and Supports Neural Differentiation in Medulloblastoma
Source: PLoS One. 2011 Sep 12;6(9):e24584. doi: 10.1371/journal.pone.0024584 (PMC3171461; doi:10.1371/journal.pone.0024584)

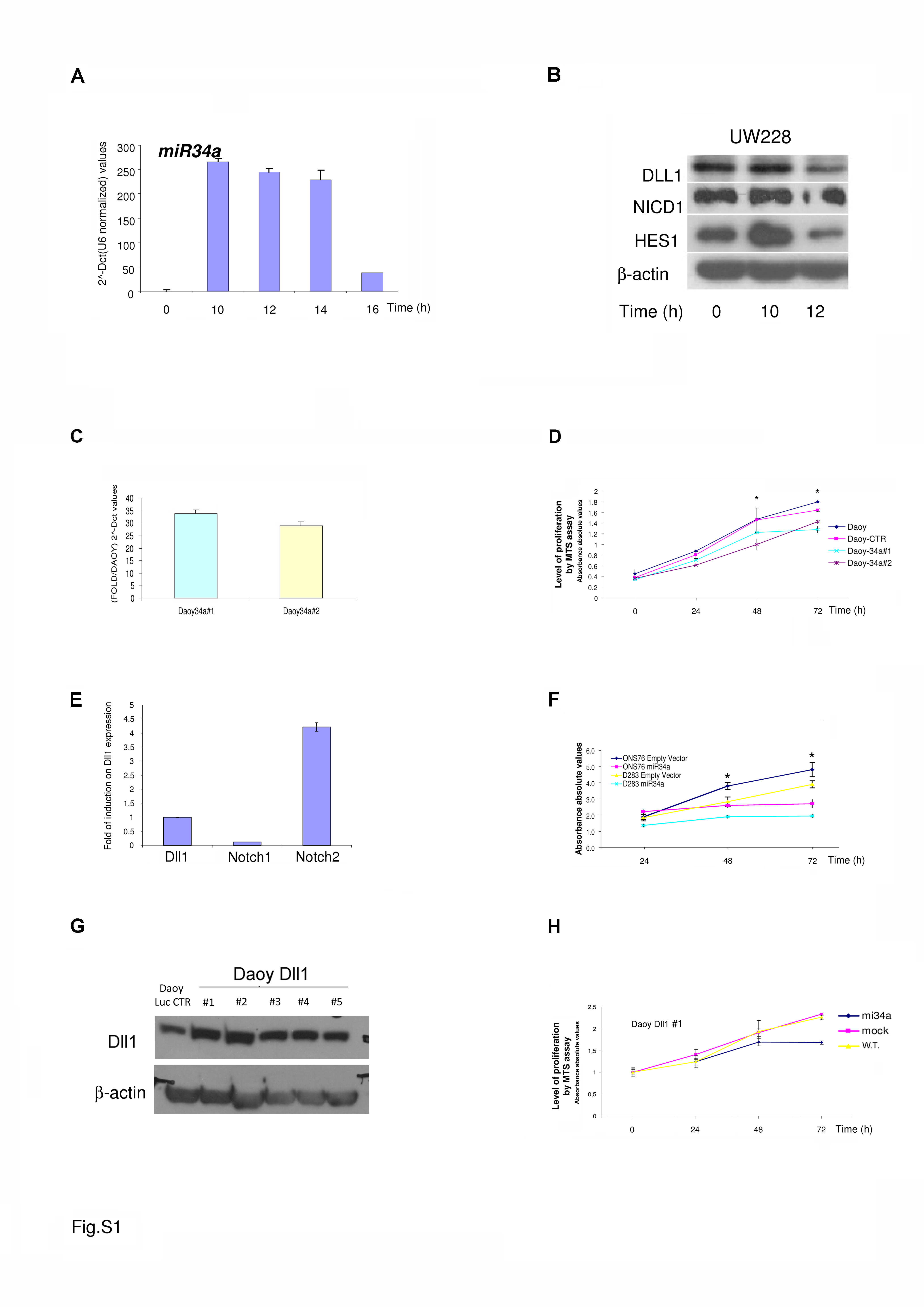

Supplement: Figure S1 — A. Real-time PCR analysis for miR-34a expression in the Daoy cell line following transfection of miR-34a at each time point from 0 h to 16 h. Real-time PCR reactions were normalized to mU6. Data are means ±standard deviation of 3 independent experiments, each carried out in triplicate. B. Representative Western blot time course performed on UW228 cells transfected with miR-34a, using an antibodies panel against: Dll1, NICD1, NICD2, Hes1 and β-actin. C. Real-time PCR analysis for miR-34a expression in Daoy miR-34a stable clones. Real-time PCR reactions were normalized to mU6. Data are means ±standard deviation of 3 independent experiments, each carried out in triplicate. D. MTS proliferation assay performed on stable Daoy miR-34a clones 1 and 2, on a stable Daoy empty vector clone and on wild-type Daoy cells. E. Real-time PCR showing Dll1, Notch1 and Notch2 expression in Daoy cells grown under conditions. Fold changes are shown respect to Dll1 expression. Real Time PCR reaction were normalized to β-Actin. Data are means ±SD from three independent experiments, each carried out in triplicate. F. MTS proliferation assay performed on ONS76 and D283 cell lines, both transfected with a vector carrying miR-34a or with an empty vector. G. Representative Western blot showing Dll1 overexpression in Daoy Dll1 stable clones 1, 2, 3,4 and 5, with respect to that of an empty vector stable clone, performed by using anti-Dll1 and anti-β-actin antibodies. H. MTS proliferation assay performed on Daoy Dll1 stable clones, infected with AdV-miR-34a or AdV-GFP-mock virus, or under basal conditions. Data are means ±SD from three independent experiments, each carried out in triplicate. (TIF) [file pone.0024584.s001.tif]

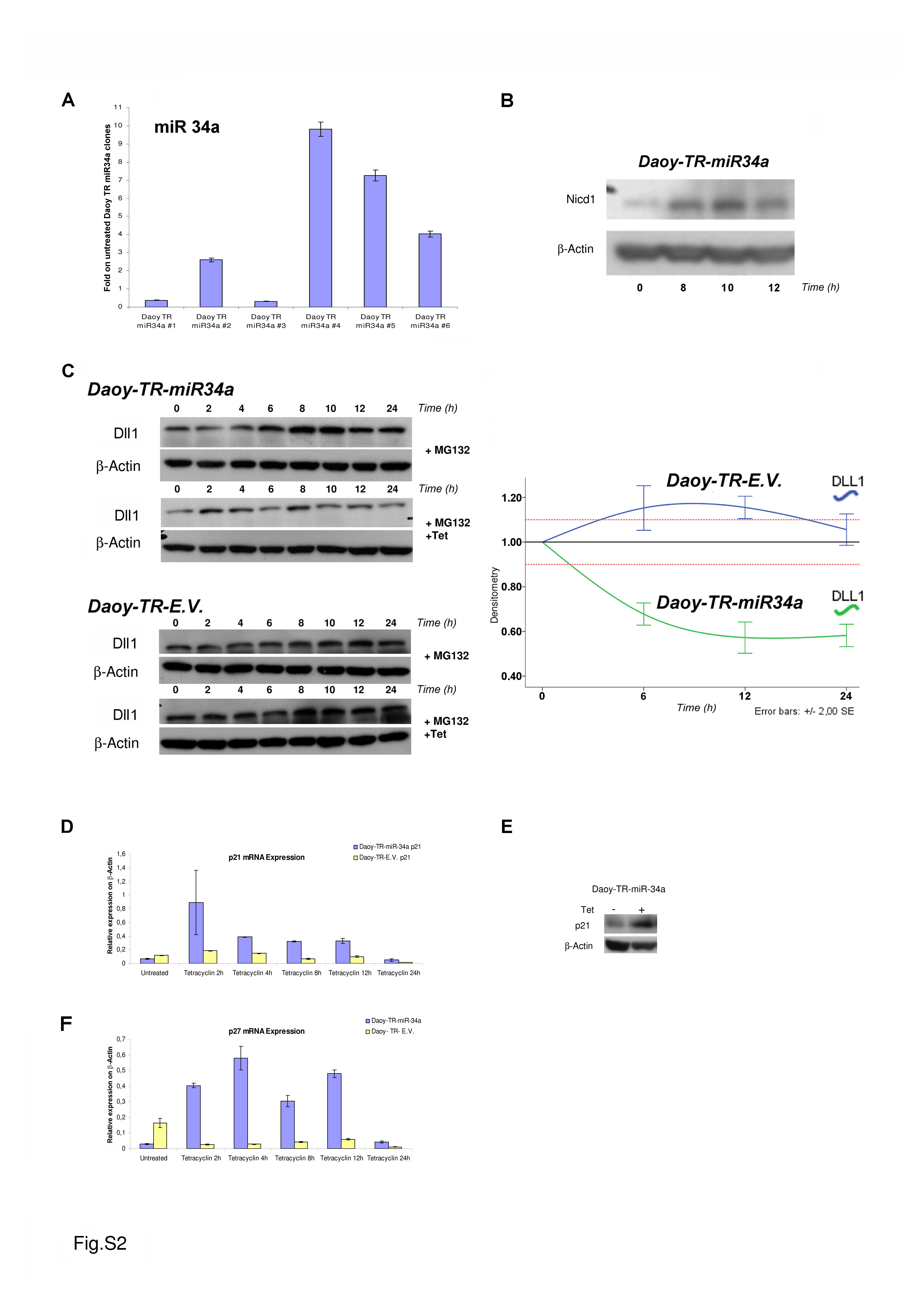

Supplement: Figure S2 — A. Real-time PCR showing miR-34a expression in Daoy–miR-34a tetracycline inducible clones (Daoy-TR-miR-34a) at 4 h from tetracycline stimulation, as normalized to sn-U6. Data are means ±SD from three independent experiments. B. Representative Western blot time courses performed on Daoy-TR-miR-34a cells with tetracycline stimulation, using an antibody panel against: NICD1 and β-actin. C. Top: Representative Western blot time courses using 2.5 µM MG132 proteasome inhibitor, performed on Daoy-TR-EV and Daoy-TR-miR-34a cells, as indicated, without and with tetracycline stimulation, using an antibody panel against: Dll1and β-actin. Bottom: Dll1 densiometric representation, as normalized to β-actin. following the tetracycline stimulated, each value was expressed as fold-stimulation over the unstimulated cells (t0). D. Real-time PCR time courses showing p21 expression in Daoy-TR-EV and Daoy-TR-miR-34a cells, treated with tetracycline. he real-time PCR reactions were normalized to β-actin. E. Representative Western blot on Daoy-TR-miR-34a cells 6h later tetracycline stimulation, using an antibody panel against: p21 and β-actin. F. Real-time PCR time courses showing p27 expression in Daoy-TR-EV and Daoy-TR-miR-34a cells, treated with tetracycline. he real-time PCR reactions were normalized to β-actin. (TIF) [file pone.0024584.s002.tif]

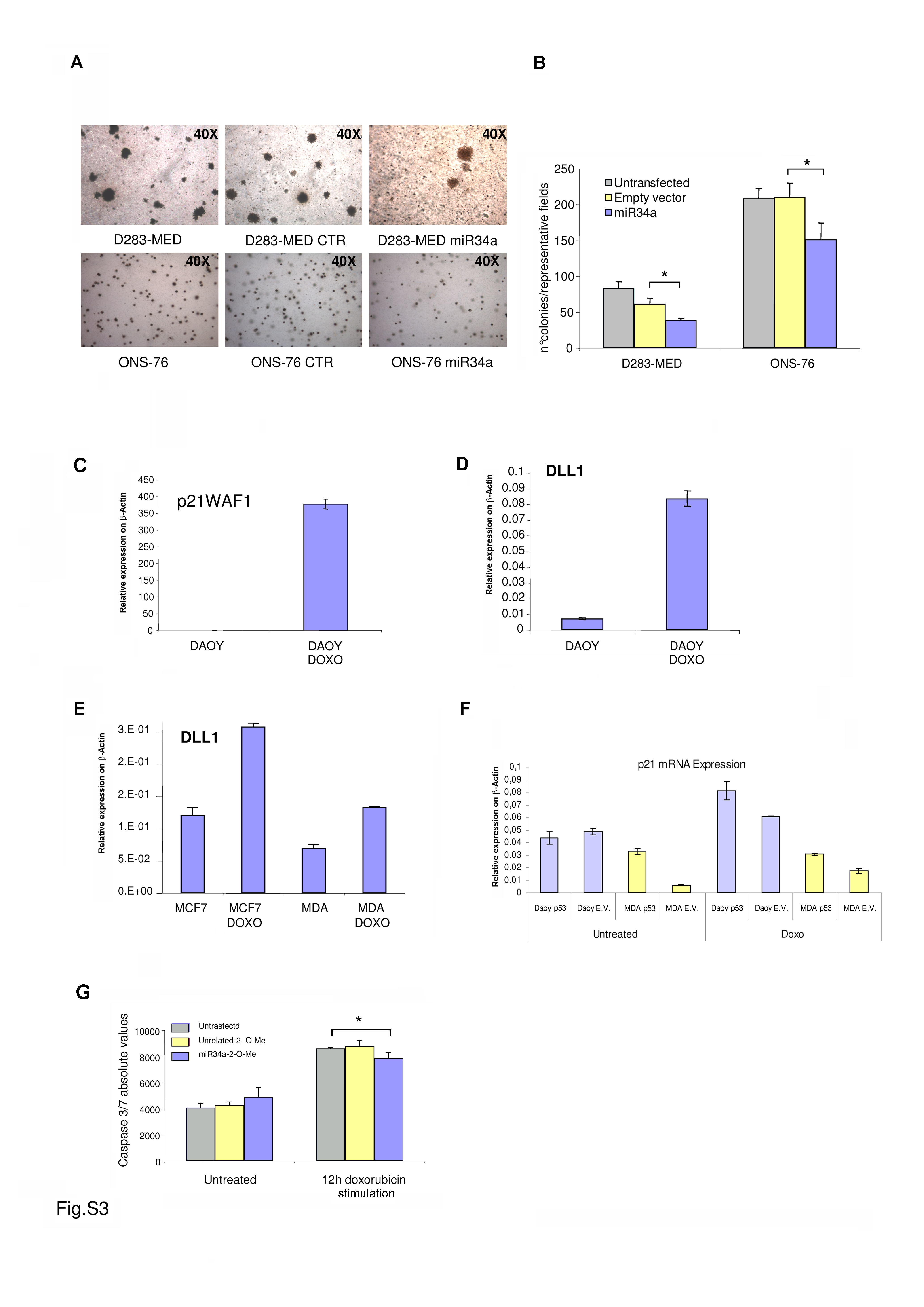

Supplement: Figure S3 — A. MiR-34a overexpression impairs soft-agar colony formation of D283-MED and ONS76 cells. Cells that received miR34a are less tumorigenic compared to untrasfected or empty vector transfected cells (p values<0.001). Representative three fields of each plate are reported on Figure S3B (cell untrasfected and empty vector or miR34a transfected) which were then counted and plotted to produce histograms represented in Figure S3B. B. Colony numbers for D283-MED and ONS-76 cells (as indicated) calculated from three representative fields of each plate, with three plates per sample for untransfected and empty vector or miR-34a transfected cells (* p<0.001). C.-D.-E. Real-time PCR analysis of induction of p21waf1 (C) and Dll1 (D, E) gene expression after 12 h of doxorubicin stimulation in MB Daoy and breast MCF7 and MDA cell lines. Data are means ±ranges of representative duplicate experiment, as normalized to β-actin expression. F. Real-time PCR showing p21 expression in Daoy, and MDA-231T cells lines transfected with p53 wt, and treated for 12h with doxorubicin, 18h later transfection. Empty vector trasfected cells were used as control. The real-time PCR reactions were normalized to β-actin. (TIF) [file pone.0024584.s003.tif]

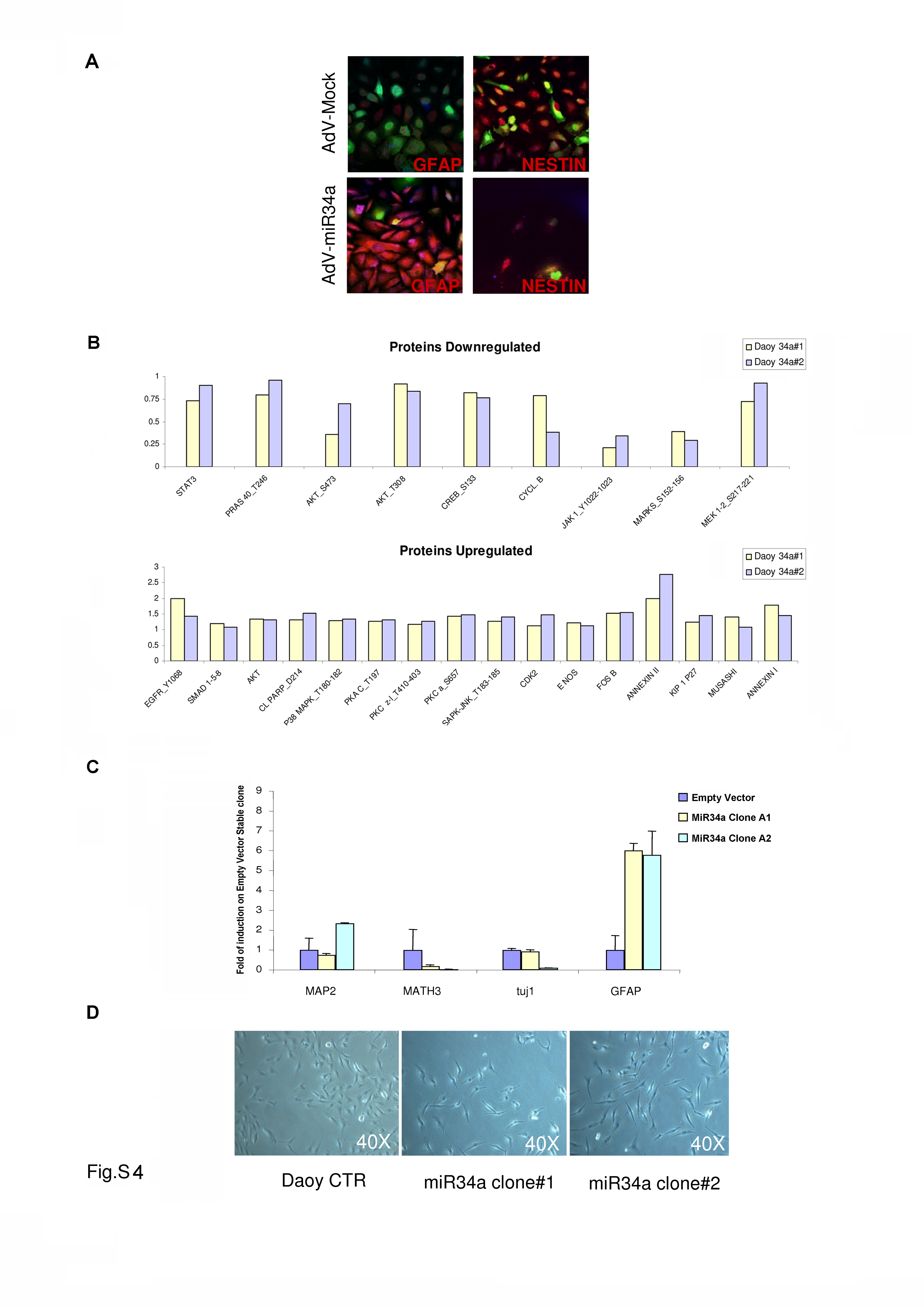

Supplement: Figure S4 — A. Representative immunofluorescence analysis of Daoy cells 48 h from infection with AdV-miR-34a or AdV-GFP-mock viruses, stained for Nestin or GFAP. B. Reverse phase proteomic array showing proteins that were down-regulated (top) and up-regulated (bottom) in miR-34a stable clones 1 and 2, compared to an empty vector stable clone. C. Real-time PCR showing the expression profiles of the neural markers MAP2, MATH3, TUJ1 and GFAP in miR-34a Daoy stable clones 1 and 2 and in an empty vector stable clone. Data are means ±ranges of representative duplicate experiment, as normalized to β-actin. D. Representative phase-contrast microscopy images (Leika DMIL, 40×0.22 magnification), showing morphological differences between an empty vector Daoy stable clone (left) and miR-34a Daoy stable clones 1 (middle) and 2 (right). The miR-34a clones show extensive neurite out-growth processes and a more differentiated phenotype. (TIF) [file pone.0024584.s004.tif]

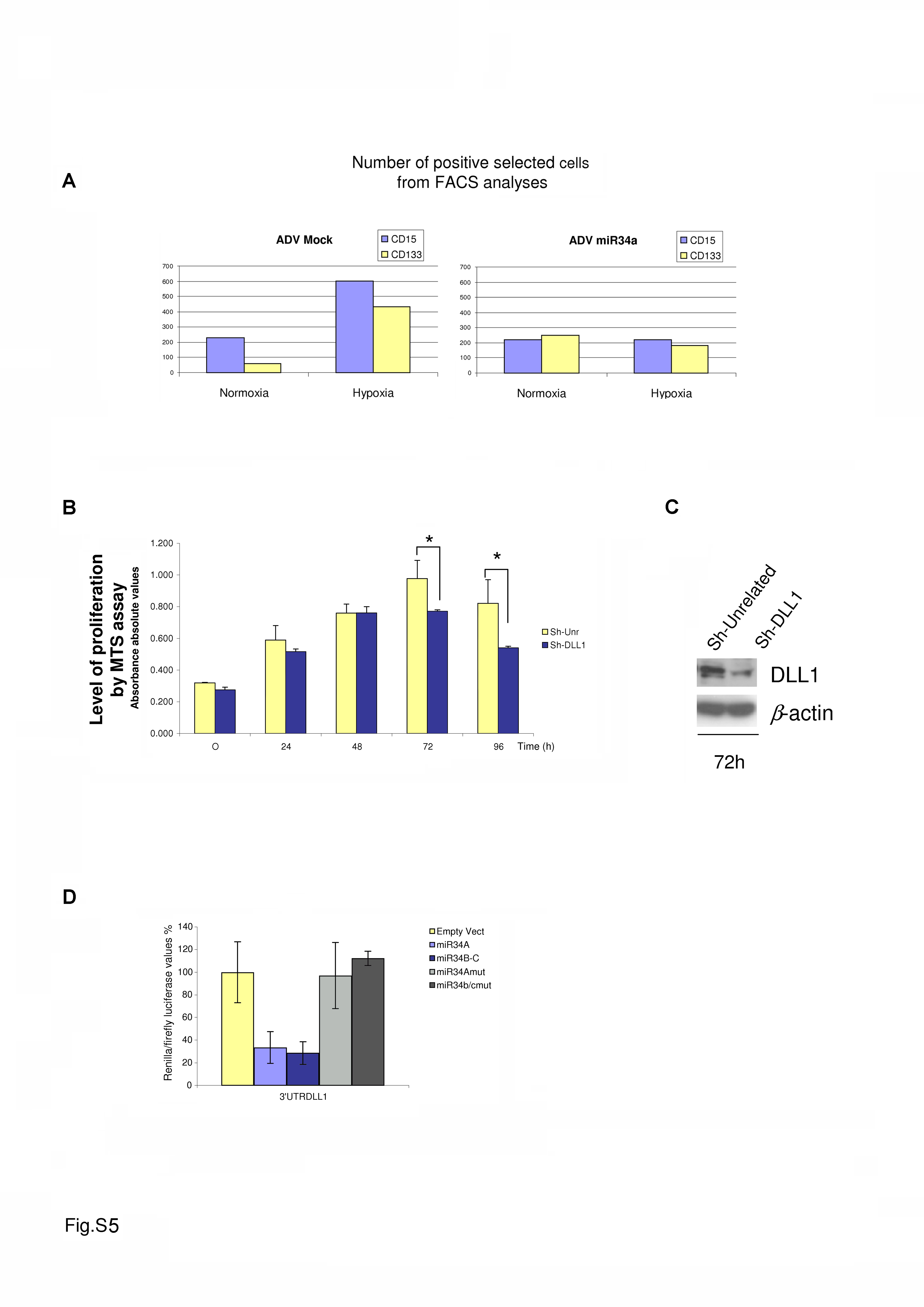

Supplement: Figure S5 — A. FACS analyses showing cell counts for CD15+ and CD133+ subpopulations in Daoy cells grown under normoxia or hypoxia conditions for 12 h, after 24 h of infection with AdV-miR-34a or AdV-mock viruses. Data are means ±SD from six independent experiments, each carried out in triplicate B. MTS proliferation assay of Daoy cells transfected with a pool of three different shRNA constructs targeting the Dll1 sequence or with an unrelated shRNA. Data are means ±SD from six independent experiments, each carried out in triplicate. Significant impairment of proliferation was seen at both 72 h (*p<0.05) and 96 h from transfection (*p<0.04). C. Representative Western blot performed using anti-Dll1 and anti-β-actin antibodies on Daoy cells at 72 h after transfection with Sh-Dll1 and with an Sh unrelated. D. Luciferase assay on Daoy cells co-transfected with Dll1 3’UTR reporter constructs and an empty vector, or with miR-34a or miR-34b, c, or with the seed-mutated miR-34a or miR34b, c. The relative luciferase activities are shown at 24 h from transfection, as normalized to the renilla luciferase activity. Data are means ±SD of six independent experiments, each performed in triplicate. The amount of transfected plasmid DNA was maintained constant by adding empty vector. (TIF) [file pone.0024584.s005.tif]

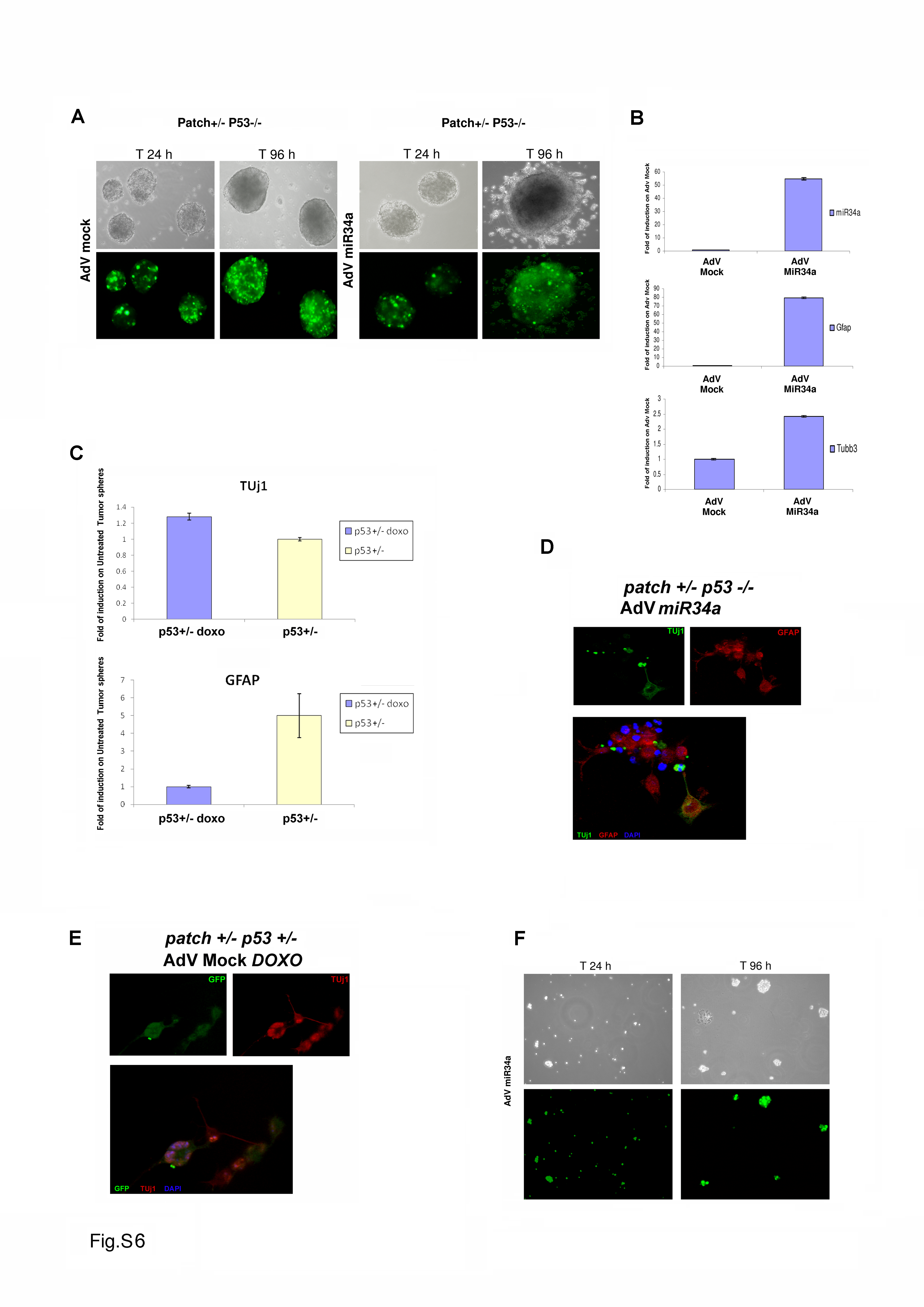

Supplement: Figure S6 — A. Confocal GFP staining on Patch +/- P53 -/- mouse tumor spheres at both 24 h and 96h from AdV-miR34a or AdV-GFP-Mock viruses infection, showing differentiating effect of AdV- miR34a. B. Real Time PCR performed on Patch +/- P53 -/- mouse tumor spheres at 48 h from infection with AdV-miR34a or AdV-GFP-Mock viruses. AdV-miR34a infected tumor spheres overexpress both miR34a and the neural differentiating markers at GFAP and Tubb3, respect to AdV-GFP-Mock infected tumor spheres. Folds of induction on AdV-GFP-Mock are shown. Data were normalized to sn-U6 and to β-actin. C. Real time PCR showing expression levels of TUj1 and GFAP in MB spheres Patch 1 +/- P53+/- treated or not with doxorubicin for 12 h, as fold-induction over untreated tumor spheres, normalized to β-actin. D. Immunofluorescence analysis of Patch 1 +/- P53+/- tumor spheres at 48h from infection with AdV-miR34a or AdV-GFP-Mock viruses, stained with anti-TUj1 or anti-GFAP antibodies. E. Immunofluorescence analysis of Patch 1 +/- P53+/- tumor spheres previously infected with AdV-GFP-Mock, treated with doxorubicin for 12 h and then stained with anti-TUj1 antibody. GFP signal from AdV-GFP-Mock virus proves cell viability in spite of doxorubicin toxicity. F. Confocal GFP staining on Patch +/- P53 -/- mouse tumor spheres previously infected with AdV-GFP-Mock and then treated with doxorubicin for 12 h. AdV- miR-34a does not exert any prodifferentiating effect at either 24 h or 96 h from infection. (TIF) [file pone.0024584.s006.tif]

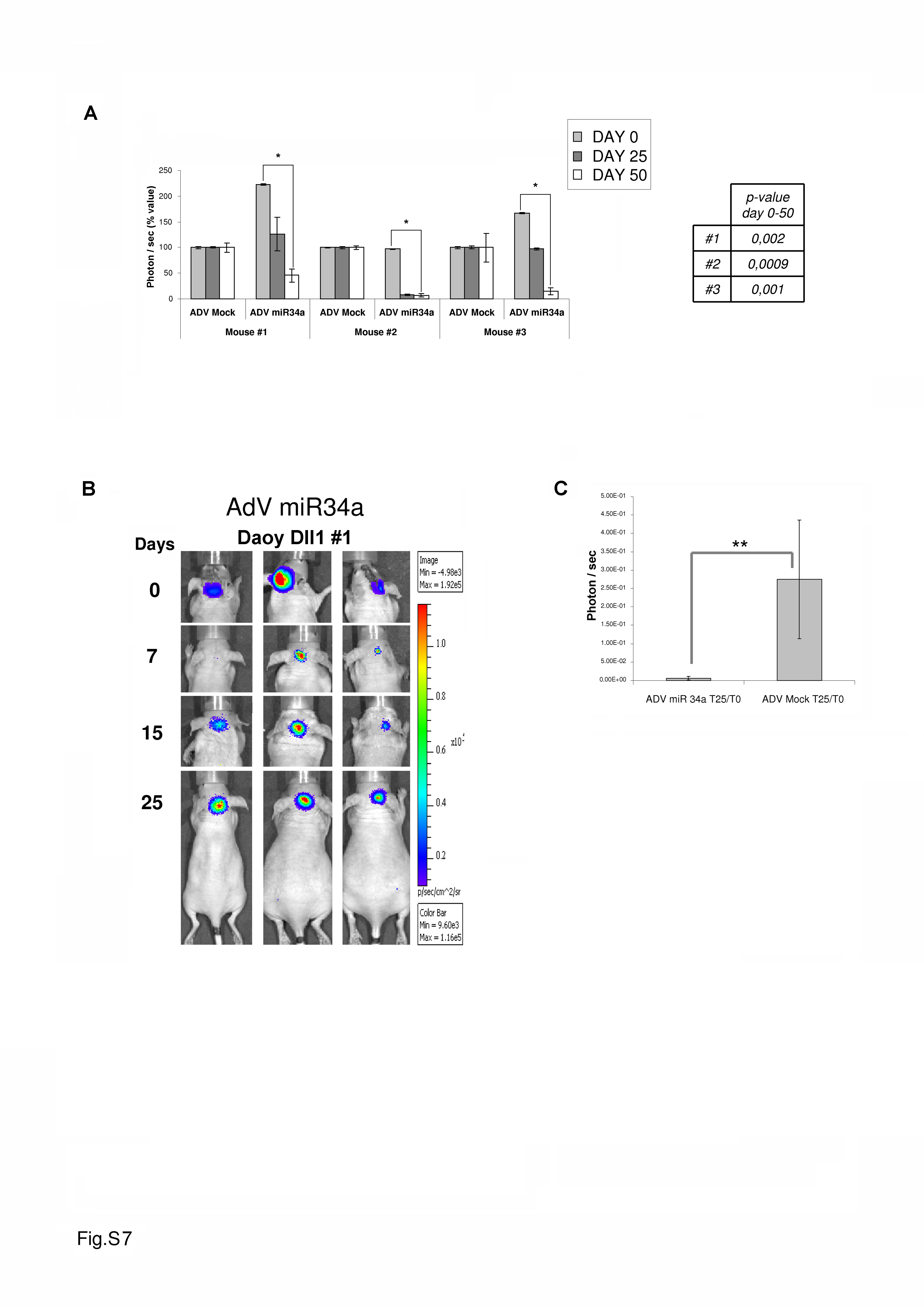

Supplement: Figure S7 — A. BLI analysis of 3 etherotopic xenografts performed with Daoy cells previously infected with AdV-miR-34a or AdV-GFP-mock viruses. BLI measurements were performed at 25 days post-implantation. P values were calculated comparing the BLI values of the AdV-miR-34a with those of the AdV-GFP-mock xenografts. B. BLI from three mice injected in the fourth cerebellar ventricle with DaoyY-Dll1 #1 Luc cells after infection with AdV-miR-34a virus. Photon emission measured at 25 days from implantation shows development and engraftment of tumor burden. C. BLI analysis of MB orthotopic xenografts of Daoy cells previously infected with AdV-miR-34a or AdV-GFP-mock viruses. The reported BLI signals are folded on that measured at t0 day. Data are mean BLI values of AdV-miR-34a and AdV-GFP-mock xenografts (n = 5 for each). (TIF) [file pone.0024584.s007.tif]

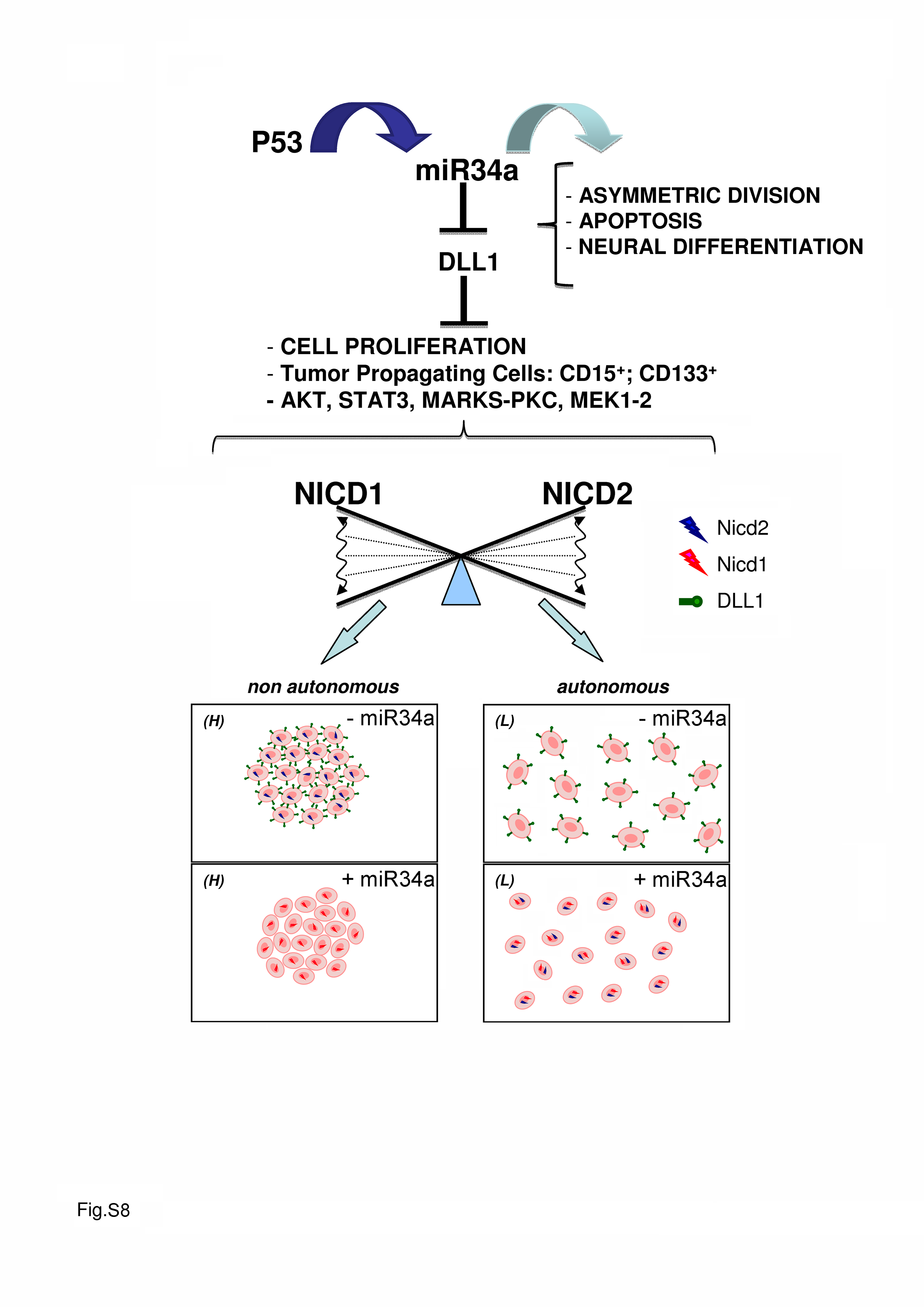

Supplement: Figure S8 — Model of the action of miR-34a upon p53 expression and regulation in MB. Cancer stem cells escape from the control of their division and go through neoplastic transformation, becoming TPCs. In MB, this process involves Notch signaling. The model takes into account the control of the p53/miR-34a/Dll1 axis with the Notch cell autonomous and cell non-autonomous pathways. We hypothesize that miR-34a increases the asymmetric division of TPCs at the expense of the symmetric self-renewing division. Within the cell autonomous context (right), miR-34a enhances Notch 2 signaling, which induces cell proliferation. Conversely, within the non-autonomous context, miR-34a enhances the pathway of Notch1, but blocks that of Notch2, which inhibits cell proliferation. (TIF) [file pone.0024584.s008.tif]
