# Supplementary material for: MiR-34a Targeting of Notch Ligand Delta-Like 1 Impairs CD15+/CD133+ Tumor-Propagating Cells and Supports Neural Differentiation in Medulloblastoma
Source: PLoS One. 2011 Sep 12;6(9):e24584. doi: 10.1371/journal.pone.0024584 (PMC3171461; doi:10.1371/journal.pone.0024584)
Supplement: Table S1 — MiR-34a targets were selected by examining the output of the indicated miRNA databases. Each database relies on different algorithms of target prediction and uses different read-out scales; e.g. PITA algorithm shows ΔΔG energetic values of the predicted miRNA/mRNA binding, so the more negative the value, the stronger the binding between the miRNA and the given site. For the 3’UTRs of Dll1, Notch1 and Jag1, more than one miR-34a-binding site was predicted. *Among the experimentally validated miR-34a targets, the Met and Bcl2 genes were chosen as references for the score values. (DOC) [file pone.0024584.s009.doc]

|  | Miranda | Target Scan | Pic Tar | PITA |
| --- | --- | --- | --- | --- |
| DLL1 | 18.13 | 90 | 14.53 | -13.98 |
| JAG1 | ----- | 77 | 4.82 | -12.78 |
| NOTCH1 | ----- | 95 | 9.64 | -14.37 |
| NOTCH2 | ----- | 68 | 5.17 | -9.93 |
| *MET | 16.82 | 88 | 8.56 | -8.16 |
| *BCL2 | ----- | 86 | 1.34 | -14.23 |

Table S1
