# Supplementary material for: MiR-34a Targeting of Notch Ligand Delta-Like 1 Impairs CD15+/CD133+ Tumor-Propagating Cells and Supports Neural Differentiation in Medulloblastoma
Source: PLoS One. 2011 Sep 12;6(9):e24584. doi: 10.1371/journal.pone.0024584 (PMC3171461; doi:10.1371/journal.pone.0024584)
Supplement: Supporting Information S1 — (DOC) [file pone.0024584.s011.doc]

**Supporting Information**

***Identification of Dll1 as miR34a target***

Through computational analyses that were performed using different algorithms and databases (mirBase, TargetScan, miRanda, PicTar and PITA) for microRNA target prediction, we noted that several predicted targets of miR-34a are key genes in Notch signaling: Dll1, Jag1, Notch1 and Notch2, two Notch ligands and two Notch receptors, respectively (table S1). To confirm that miR-34a can down-regulate the Dll1 protein in different MB cell lines, we transiently transfected UW228 cells with a miR-34a expressing vector and performed a time course with Western blotting analysis to check both Dll1 and Hes1. MiR-34a overexpression led to a decrease in the protein levels of both Dll1 and Hes1 at 12 h from transfection (Fig. S1B). To analyze the effects of miR-34a overexpression on cell proliferation, we carried out an MTS assay on the Daoy miR-34a stable clones 1 and 2, on a Daoy empty vector stable clone, and on wild-type Daoy cells. The proliferation rates of both of these Daoy miR-34a stable clones was impaired compared to the Daoy empty vector stable clone (Fig. S1D). Therefore, to attribute the antiproliferative effects of miR-34a to Dll1 down-regulation, we generated Daoy cells overexpressing the hDll1 cDNA devoid of the 3’UTR, and so not targeted by miR-34a. Using Western blotting to detect the Dll1 protein levels, we chose clone1 among the several Daoy Dll1 clones generated (Fig. S1H). We then performed an MTS assay on these cells upon transfection with a miR-34a expressing vector or with an empty vector, and under basal conditions. Dll1 overexpression did not rescue the antiproliferative effects of miR-34a (Fig. S1G). The explanation for this result relates to the off-target effects of miR-34a, which besides targeting Dll1, also down-regulates other genes that are involved in cell proliferation.

**A specific Dll1 shRNA impairs cell proliferation**

Previous studies have demonstrated that the soluble dominant-negative form of Dll1 inhibits cell proliferation in Daoy and D283-MED cells (Hallahan et al., 2004). To determine whether Dll1 down-regulation inhibits cell proliferation, we transfected Daoy cells with a Dll1-shRNA construct; after 72 h, there was a significant reduction in the proliferation rate, as well as after miR-34a transfection (Fig. S5A, B). These findings suggest that in MB cells, miR-34a performs its antiproliferative effect by down-regulating Dll1.

To further confirm that miR-34a has a central role in apoptosis, we measured caspase 3/7 activities in Daoy cells transfected or not with miR-34a-2-O-Me or with an unrelated-2-O-Me, and stimulated or not with doxorubicin, a genotoxic agent. Without doxorubicin, caspase activation of transfected cells resembled that of untransfected cells. In the presence of doxorubicin, instead, caspase activation of miR-34a-2-O-Me transfected cells was significantly lower than that of the unrelated-2-O-Me transfected cells, and of untransfected cells (Fig. S5C). These results show that in Daoy cells, the silencing of miR-34a softens the proapoptotic effect of doxorubucin.

As already explained in the main test, doxorubicin stimulation activates p53, induces miR-34a expression, and finally down-regulates the Dll1 protein (Fig. 4E). We asked if the negative regulation of Dll1 at the protein level was balanced by an increase in the expression of Dll1 mRNA. We used real-time PCR analysis to measure *DLL1* gene expression upon doxorubicin stimulation in the MB Daoy (Fig. S3D) and breast cancer MCF7 and MDA235T cell lines (Fig. S3E). Doxorubicin treatment increased Dll1 expression in all of the cell lines tested. As evidence of p53 activation, we performed real-time PCR to analyze the expression of p21waf1, the main target of p53, in Daoy cells treated or not with doxorubicin (Fig. S3C). As described in the main body of the text, in these Daoy cells, we rescued the activation of caspase 3/7 induced by miR-34a overexpression (Fig. 4C), by transfecting the wild type cDNA of murine Dll1.

**MiR-34a impacts on all the genome protein** phosphorylation

We wandered what was the impact of miR-34a on protein output. We thus used reverse phase proteomic array technology to detect proteins that were up-regulated or down-regulated, and that changed as a consequence of miR-34a overexpression (Fig. S4B). This analyses helped us to understand how miR-34a overexpression influences the biology and cancerogenic properties of stem cells. In Daoy miR-34a stable clones, the level of PTEN phosphorylated on S380 was higher than that of the empty-vector stable clone. PTEN and p53 negatively regulate neural stem-cell self-renewal; moreover, their deficiency decreases apoptosis in the tumor spheres (Zheng et al., 2008). We also observed up-regulation of the Src family phosphorylation status, on Y416. The activity of Src proteins is regulated through phosphorylation on the Y416-equivalent site, which activates Src mainly via autophosphorylation, and on the Y527-equivalent site, which inhibits Src via C-terminal Src kinase (Csk) and Csk homologous kinase phosphorylation (Chiang and Sefton, 2000; Roskoski, 2005). The phosphorylation status at Src Y527 regulates the phosphorylation at Y416 too (Roskoski, 2004; Sun et al., 1998). Determining how miR-34a influences protein phosphorylation in MB will be an issue to be approached in future studies.

On the other hand, in miR-34a over-expressing cells, there was reduced myristoylated alanine-rich C-kinase substrate (MARCKS; the main intracellular substrate for protein kinase C [PKC] (for phosphorylation on S152 and S158)). MARCKS is implicated in brain development, macrophage activation, neuro-secretion and growth-factor-dependent mitogenesis. It probably acts as a regulatory cross-bridge between actin and the plasma membrane, and it is also involved in the modulation of the actin cross-linking activity through calmodulin phosphorylation. MARCKS potentially represents a convergence point between the calcium–calmodulin and the signal transduction pathways of PKC, both of which are implicated in the regulation of the actin cytoskeleton (Blackshear, 1993). Furthermore in miR-34a-over-expressing cells, there was reduced phosphorylation status of MEK 1/2 (on S217, S221). The mitogen-activated protein kinase (MAPK) cascade is the key signaling pathway in the regulation of cell survival, proliferation and differentiation (Roberts and Der, 2007). As MEK and MAPK show a decreased phosphorylation status in miR-34a-over-expressing cells. The miR-34a ability to impair both MEK and MARCKS activities is a finding of a great interest in the search for new anti-cancer therapies.

A list of the proteins that were up-regulated or down-regulated with statistical significance in the Daoy miR-34a stable clones is given in Figure S4B. Using Western blotting, we validated the differences in the levels of some of the proteins mentioned (Fig. 5E).

**Mir-34a influences the differentiation process in both neural and glial cells.**

With respect to the empty vector stable clone, the Daoy miR-34a stable clones 1 and 2 have a more differentiated phenotype that is characterised by neurite sprouting (Fig. S4D). Of note, Daoy cells have been previously reported to specifically express glial-specific proteins under different conditions, such as phenylbutyrate treatment or epidermal growth factor (EGF) stimulation (Li et al., 2004; Shen et al., 2001). Therefore we asked whether miR-34a overexpression influences the differentiation rate of Daoy cells. Using real-time PCR, we analyzed the expression profiles of some neural markers in these Daoy miR-34a clones, as compared to the empty vector stable clone. There were no significant differences in the expression of the neuronal markers TUj1, MATH3 and MAP2; however, the glial marker GFAP was highly expressed in both of these Daoy miR-34a clones, as compared to the empty vector stable clone (Fig. S4C).

As with the Patch1^+/-^ p53^+/-^ mouse MB spheres (Fig. 6A-C), Patch1^+/-^ p53^-/-^ mouse MB spheres showed a differentiated phenotype at 96 h from infection with the AdV-GFP-miR-34a virus (Fig. S6A). Real-time PCR analyses (Fig. S6B) and immunofluorescence (Fig. S6D) showed that in these cells, miR-34a has its antiproliferative effect, by increasing the expression of both Tubb3 and GFAP. Thus, we performed real-time PCR analysis to determine whether in the Patch1^+/-^ p53^+/-^ mouse MB spheres, doxorubicin treatment increases expression of neural markers. Our data show that doxorubicin enhances the expression of TUj1, but decreases that of GFAP (Fig. S6C).

Doxorubicin treatment promotes differentiation in the Patch1^+/-^ p53^+/-^ MB spheres (Fig. 6B). To support these data in a control experiment, we stimulated Patch1^+/-^ p53^+/-^ MB spheres that had been previously infected with AdV-GFP-mock virus with doxorubicin. The prodifferentiating effects of doxorubicin were confirmed here (Fig. S6E). Moreover, the GFP signal from the AdV-GFP-mock virus showed that the differentiated cells maintained their viability in spite of doxorubicin toxicity.

As indicated in the main text, the AdV-miR-34a virus only efficiently infects those cells that are located into the most external region of these tumor spheres (Fig. 6C). The external cells overexpressing miR-34a promote differentiation of the inner cells. We aimed to investigate this by infecting previously dissociated Patch1^+/-^ p53^+/-^ MB spheres with AdV-GFP-miR-34a virus. These infected cells did not differentiate, and underwent apoptosis instead (Fig. S6F). The explanation for this is that all of the cells received the same number of viral particles, so there were not any uninfected cells which could get prodifferentiating stimuli from the efficiently infected cells. Consequently the proapoptotic effect of miR-34a was exerted at the expense of the prodifferentiating effect, which was bypassed.

***In-vivo* Dll1 restoration promotes tumor engraftment of Adv-miR-34a-infected cells.**

To demonstrate not only *in vitro*, but also *in vivo* that the antitumorigenic effects of miR-34a depend on the down-regulation of Dll1, we implanted Daoy Dll1#1 cells that were pre-infected with AdV-GFP-miR-34a into the cerebellum of nude mice. As expected, at 25 days post-implantation, there was no impairment of tumorigenesis. These data show that Dll1 restoration can rescue *in vivo* the anti-engraftment effects of miR-34a.

DISCUSSION

There is increasing evidence that suggests that miRNAs are dysregulated in several neurological disorders and tumors that arise in the central nervous system. The developing central nervous system expresses one of the richest diversities of miRNAs of any tissue (Kapsimali et al., 2007; Krichevsky et al., 2006; Miska et al., 2004; Sempere et al., 2004). Several miRNAs have already been shown to contribute to cell proliferation and specification (Schwamborn et al., 2009; Zhao and Liu, 2009). Indeed, one potential drawback in the therapeutic use of miRNAs in the nervous system is possible alterations of the normal physiology in addition to the disease pathology, due to the many potential targets of any given miRNA.

Endogenous miRNAs regulate hundreds of targets by mRNA degradation or by translational repression of protein production (Baek et al., 2008; Lim et al., 2005; Selbach et al., 2008). The viral delivery method of miRNAs allows for more precise spatially targeted therapies through tissue-specific viral injections, potentially avoiding some of the pitfalls of systemically delivered oligonucleotides. Reports of toxicity resulting from viral shRNA delivery have recently emerged (Grimm and Kay, 2007; McBride et al., 2008). This toxicity was reported to cause cell death in both the striatum and cerebellum when delivered using an adeno-associated virus (Boudreau et al., 2009). One additional pitfall would come if the miRNA delivery is substantial and consistent with time, thus resulting in interference with endogenous RNA interference processes and competition for components of the RISC pathway. In our analyses, we created an adenovirus carrying a pre-miRNA, miR-34a, hairpin structure under a strong CMV promoter, creating an artificial “miRNA scaffold”, which thus showed no sign of toxicity. For the use of these technologies, see also Castanotto et al. (2007).

**Supplemental Figure legends**

**FigureS1 A.** Real-time PCR analysis for miR-34a expression in the Daoy cell line following transfection of miR-34a at each time point from 0 h to 16 h. Real-time PCR reactions were normalized to mU6. Data are means ±standard deviation of 3 independent experiments, each carried out in triplicate. **B.** Representative Western blot time course performed on UW228 cells transfected with miR-34a, using an antibodies panel against: Dll1, NICD1, NICD2, Hes1 and β-actin. **C.** Real-time PCR analysis for miR-34a expression in Daoy miR-34a stable clones. Real-time PCR reactions were normalized to mU6. Data are means ±standard deviation of 3 independent experiments, each carried out in triplicate. **D.** MTS proliferation assay performed on stable Daoy miR-34a clones 1 and 2, on a stable Daoy empty vector clone and on wild-type Daoy cells. **E.** Real-time PCR showing Dll1, Notch1 and Notch2 expression in Daoy cells grown under conditions. Fold changes are shown respect to Dll1 expression. Real Time PCR reaction were normalized to β-Actin. Data are means **±**SD from three independent experiments, each carried out in triplicate. **F**. MTS proliferation assay performed on ONS76 and D283 cell lines, both transfected with a vector carrying miR-34a or with an empty vector. **G.** Representative Western blot showing Dll1 overexpression in Daoy Dll1 stable clones 1, 2, 3 ,4 and 5, with respect to that of an empty vector stable clone, performed by using anti-Dll1 and anti-β-actin antibodies. **H.** MTS proliferation assay performed on Daoy Dll1 stable clones, infected with AdV-miR-34a or AdV-GFP-mock virus, or under basal conditions. Data are means **±**SD from three independent experiments, each carried out in triplicate.

**Figure S2 A.** Real-time PCR showing miR-34a expression in Daoy–miR-34a tetracycline inducible clones (Daoy-TR-miR-34a) at 4 h from tetracycline stimulation, as normalized to sn-U6. Data are means **±**SD from three independent experiments.**B.** Representative Western blot time courses performed on Daoy-TR-miR-34a cells with tetracycline stimulation, using an antibody panel against: NICD1 and β-actin. **C.** Top: Representative Western blot time courses using 2.5 μM MG132 proteasome inhibitor, performed on Daoy-TR-EV and Daoy-TR-miR-34a cells, as indicated, without and with tetracycline stimulation, using an antibody panel against: Dll1and β-actin. Bottom: Dll1 densiometric representation, as normalized to β-actin. following the tetracycline stimulated, each value was expressed as fold-stimulation over the unstimulated cells (t0). **D.** Real-time PCR time courses showing p21 expression in Daoy-TR-EV and Daoy-TR-miR-34a cells, treated with tetracycline. he real-time PCR reactions were normalized to β-actin. **E.** Representative Western blot on Daoy-TR-miR-34a cells 6h later tetracycline stimulation, using an antibody panel against: p21 and β-actin. **F.** Real-time PCR time courses showing p27 expression in Daoy-TR-EV and Daoy-TR-miR-34a cells, treated with tetracycline. he real-time PCR reactions were normalized to β-actin.

**Figure S3. A.** MiR-34a overexpression impairs soft-agar colony formation of D283-MED and ONS76 cells. Cells that received miR34a are less tumorigenic compared to untrasfected or empty vector transfected cells (p values < 0.001). Representative three fields of each plate are reported on Figure S3B (cell untrasfected and empty vector or miR34a transfected) which were then counted and plotted to produce histograms represented in Figure S3B. **B.** Colony numbers for D283-MED and ONS-76 cells (as indicated) calculated from three representative fields of each plate, with three plates per sample for untransfected and empty vector or miR-34a transfected cells (* p < 0.001). **C.-D.-E.** Real-time PCR analysis of induction of p21waf1 (C) and Dll1 (D, E) gene expression after 12 h of doxorubicin stimulation in MB Daoy and breast MCF7 and MDA cell lines. Data are means ±ranges of representative duplicate experiment, as normalized to β-actin expression. **F.** Real-time PCR showing p21 expression in Daoy, and MDA-231T cells lines transfected with p53 wt, and treated for 12h with doxorubicin, 18h later transfection. Empty vector trasfected cells were used as control. The real-time PCR reactions were normalized to β-actin.

**Figure S4. A.** Representative immunofluorescence analysis of Daoy cells 48 h from infection with AdV-miR-34a or AdV-GFP-mock viruses, stained for Nestin or GFAP. **B.** Reverse phase proteomic array showing proteins that were down-regulated (top) and up-regulated (bottom) in miR-34a stable clones 1 and 2, compared to an empty vector stable clone.

**C.** Real-time PCR showing the expression profiles of the neural markers MAP2, MATH3, TUJ1 and GFAP in miR-34a Daoy stable clones 1 and 2 and in an empty vector stable clone. Data are means ±ranges of representative duplicate experiment, as normalized to β-actin. **D.** Representative phase-contrast microscopy images (Leika DMIL, 40× 0.22 magnification), showing morphological differences between an empty vector Daoy stable clone (left) and miR-34a Daoy stable clones 1 (middle) and 2 (right) . The miR-34a clones show extensive neurite out-growth processes and a more differentiated phenotype.

**Figure S5. A.** FACS analyses showing cell counts for CD15^+^ and CD133^+^ subpopulations in Daoy cells grown under normoxia or hypoxia conditions for 12 h, after 24 h of infection with AdV-miR-34a or AdV-mock viruses. Data are means **±**SD from six independent experiments, each carried out in triplicate **B.** MTS proliferation assay of Daoy cells transfected with a pool of three different shRNA constructs targeting the Dll1 sequence or with an unrelated shRNA. Data are means **±**SD from six independent experiments, each carried out in triplicate. Significant impairment of proliferation was seen at both 72 h (*p < 0.05) and 96 h from transfection (*p < 0.04). **C.** Representative Western blot performed using anti-Dll1 and anti-β-actin antibodies on Daoy cells at 72 h after transfection with Sh-Dll1 and with an Sh unrelated. **D.** Luciferase assay on Daoy cells co-transfected with Dll1 3’UTR reporter constructs and an empty vector, or with miR-34a or miR-34b, c, or with the seed-mutated miR-34a or miR34b, c. The relative luciferase activities are shown at 24 h from transfection, as normalized to the renilla luciferase activity. Data are means ±SD of six independent experiments, each performed in triplicate. The amount of transfected plasmid DNA was maintained constant by adding empty vector.

**Figure S6. A.** Confocal GFP staining on Patch +/- P53 -/- mouse tumor spheres at both 24 h and 96h from AdV-miR34a or AdV-GFP-Mock viruses infection, showing differentiating effect of AdV- miR34a. **B.** Real Time PCR performed on Patch +/- P53 -/- mouse tumor spheres at 48 h from infection with AdV-miR34a or AdV-GFP-Mock viruses. AdV-miR34a infected tumor spheres overexpress both miR34a and the neural differentiating markers at GFAP and Tubb3, respect to AdV-GFP-Mock infected tumor spheres. Folds of induction on AdV-GFP-Mock are shown. Data were normalized to sn-U6 and to β-actin. **C.** Real time PCR showing expression levels of TUj1 and GFAP in MB spheres Patch 1 +/- P53^+^/- treated or not with doxorubicin for 12 h, as fold-induction over untreated tumor spheres, normalized to β-actin. **D.** Immunofluorescence analysis of Patch 1 +/- P53^+^/- tumor spheres at 48h from infection with AdV-miR34a or AdV-GFP-Mock viruses, stained with anti-TUj1 or anti-GFAP antibodies. **E.** Immunofluorescence analysis of Patch 1 +/- P53^+^/- tumor spheres previously infected with AdV-GFP-Mock, treated with doxorubicin for 12 h and then stained with anti-TUj1 antibody. GFP signal from AdV-GFP-Mock virus proves cell viability in spite of doxorubicin toxicity. **F.** Confocal GFP staining on Patch +/- P53 -/- mouse tumor spheres previously infected with AdV-GFP-Mock and then treated with doxorubicin for 12 h. AdV- miR-34a does not exert any prodifferentiating effect at either 24 h or 96 h from infection.

**Figure S7. A.** BLI analysis of 3 etherotopic xenografts performed with Daoy cells previously infected with AdV-miR-34a or AdV-GFP-mock viruses. BLI measurements were performed at 25 days post-implantation. P values were calculated comparing the BLI values of the AdV-miR-34a with those of the AdV-GFP-mock xenografts. **B.** BLI from three mice injected in the fourth cerebellar ventricle with DaoyY-Dll1 #1 Luc cells after infection with AdV-miR-34a virus. Photon emission measured at 25 days from implantation shows development and engraftment of tumor burden. **C.** BLI analysis of MB orthotopic xenografts of Daoy cells previously infected with AdV-miR-34a or AdV-GFP-mock viruses. The reported BLI signals are folded on that measured at t0 day. Data are mean BLI values of AdV-miR-34a and AdV-GFP-mock xenografts (n = 5 for each).

**Figure S8. Model of the action of miR-34a upon p53 expression and regulation in MB.** Cancer stem cells escape from the control of their division and *go through* neoplastic transformation, becoming TPCs. In MB, this process involves Notch signaling. The model takes into account the control of the p53/ miR-34a/ Dll1 axis with the Notch cell autonomous and cell non-autonomous pathways. We hypothesize that miR-34a increases the asymmetric division of TPCs at the expense of the symmetric self-renewing division. Within the cell autonomous context (right), miR-34a enhances Notch 2 signaling, which induces cell proliferation. Conversely, within the non-autonomous context, miR-34a enhances the pathway of Notch1, but blocks that of Notch2, which inhibits cell proliferation.

**Table S1.** MiR-34a targets were selected by examining the output of the indicated miRNA databases. Each database relies on different algorithms of target prediction and uses different read-out scales; e.g. PITA algorithm shows ΔΔG energetic values of the predicted miRNA/mRNA binding, so the more negative the value, the stronger the binding between the miRNA and the given site. For the 3’UTRs of Dll1, Notch1 and Jag1, more than one miR-34a-binding site was predicted. *Among the experimentally validated miR-34a targets, the Met and Bcl2 genes were chosen as references for the score values.

**Movie S1.** Tumor spheres isolated from Patch1^+/-^ P53^-/-^ mice infected with AdV-miR-34a show sign of induction of differentiation only when they are not dissociated before the infection. At this time, the infected cells remain in contact with each other and are subjected to Notch signaling via the cell non-autonomous pathway. A time-series of phase-contrast brightfield images of the medullpspheres were acquired with an A-Plan Ph1 10× objective for a total time of 72 h, using a Zeiss Axiovert 200M microscope equipped with an Okolab WJ CO_2_ Microscope Stage Incubator, for controlling the temperature and the %CO_2_. The time delay between individual images used here was 15 min.

**Materials and Methods**

**MiR 34a target prediction**

MiR-34a targets were selected by examining predicted targets from mirBase, TargetScan, miRanda, PicTar and PITA. Each database relies on different algorithms of target prediction and uses different read-out scales*.* Potential gene targets were chosen based on the presence in at least two of all of the predicted algorithms used in the analyses. See also Figure S1A, B and Figure legends.

**Time-course experiments**

Two different transfections were performed in Daoy, D283-MED and UW228 cells prepared in 6-well plates, to obtain the following time points: first transfection, from 6 h to 14 h; second transfection, from 16 h to 20 h. The cells were transfected at 80% confluence using the *Trans*IT-LT1 reagent (Mirus, from Cambridge Bioscience Limited, UK), using 7 μl reagent and 2.5 μg DNA per well. At each time point, the cells were harvested and lysed using RIPA buffer or TRIZOL reagent, according to the manufacturer instructions.

**Stable clone selection**

Daoy cells were transfected using *Trans*IT-LT1 reagent (Mirus), according to the manufacturer instructions. Briefly, the cells were plated in 10-cm dishes for 60% confluence the following day, when the culture medium was replaced with 16 ml complete growth medium, and a mix of 45 μl *Trans*IT-LT1 reagent and 15 μg DNA was added to the cells. From 48 h, the cells were maintained in complete growth medium containing 0.3 μg/ml neomycin (GIBCO). These were followed for at least three weeks for single clone growth (clone#1 and #2).

**MTS cell proliferation assay**

For the stable clones, 2,000 viable cells were plated in 96-well plates and left overnight. MTS proliferation assays were then performed using the CellTiter 96® AQueous Non-Radioactive Cell Proliferation Assay (Promega, Milan, Italy), according to the manufacturer procedure. Briefly, at the indicated times, the cell-culture medium was replaced with complete growth medium containing a tetrazolium salt compound. After 2 h the absorbance at 490 nm was measured using an EnVision 2102 multilabel reader (PerkinElmer, Waltham, USA). For transiently transfected cells, the cells in six-well plates were transfected as previously described (Garzia et al., 2009). Twenty-four hours after transfection, 2,000 viable cells were plated into 96-well plates, with the MTS cell proliferation assays performed as above.

**Apoptosis analysis**

Caspase activity was measured in accordance with the manufacturer’s procedure (BD Biosciences). Briefly, for D283-MED and ONS-76 3,000 viable cells plated in 96-well plates were transfected using the *Trans*IT-LT1 reagent (Mirus, from Cambridge Bioscience Limited, UK). After the indicated times, the cells were lysed by adding an equal amount of 2× lysis buffer (60 mM Tris, pH 7, 300 mM NaCl, 2% Triton X100, 2% glycerol) to the growth medium. Ten μl of cell lysate was incubated for 1 h at 37 °C in the dark, in 96-well plates (Corning from Celbio, Milan , Italy) with 90 μl reaction mix of: 10 μl reaction buffer (20 mM Hepes, 10 mM NaCl, 1 mM EDTA, 0.1% CHAPS), 50 μl 20% sucrose, 1 μl 1 M dithiothreitol, 0.2 μl Ac-DEVD-AFC caspase 3/7 fluorogenic substrate (Enzo Life Science Biomol, Plymouth Meeting, PA, USA), made up to 90 μl with water. After a 1-h incubation in the dark, the fluorescence emission at 505 nm was measured using an EnVision 2102 multilabel reader (PerkinElmer, Waltham, USA).

**Soft-agar colony formation assay.**

D283-MED and ONS-76 cells were transfected with the miR-34a-expressing vector or the empty control vector. After 24 h, 10,000 cells were plated in the appropriate medium in 0.35% agar over a 0.5% agar base. Three plates were prepared for each transfection (miR-34a and empty vector) and for untransfected cells. The medium was replaced every week. After eight weeks the colonies were counted and the data plotted.

**Reverse-phase protein microarray**

The cells were lysed in lysis buffer (Tissue Protein Extraction Reagent; Pierce) with protein inhibitors (300 mM NaCl [Sigma], 1 mM orthovanadate [Sigma], 200 mM Pefabloc [Roche, Palo Alto, CA, USA)], and 1 μg/ml aprotinin, 5 mg/ml pepstatin A, and 1 mg/ml leupeptin [Sigma, Milan, Italy)]). The cell lysates were diluted to 1 mg/ml protein in 2× Tris-glycine SDS sample buffer (Invitrogen Life Technologies) plus 5% β-mercaptoethanol. Reverse phase protein microarrays were assayed in duplicate with whole-cell-lysate proteins, as described by Liotta et al. (2003) and Paweletz et al. (2001). Briefly the lysates were assayed on glass-backed nitrocellulose array slides (FAST slides, Whatman Schleicher & Schuell, GE Healthcare) using an Aushon 2470 arrayer (Aushon Biosystems, Billerica, USA). Each lysate was assayed as a dilution curve that represented undiluted lysate, plus 1:2, 1:4 and 1:8 dilutions, and negative-control dilutions. A431, A431+EGF, HeLa, HeLa+pervanadate, HeLa+TPA, Jurkat, NIH-3T3 and NIH-3T3+UV (BD Pharmingen, San Diego, CA) cell lysates were assayed on each array, for quality-control assessment.

The protein microarray slides were prepared for immunostaining by washing with phosphate-buffered saline (PBS) without calcium or magnesium, and blocking with I-Block in PBS plus 0.5% Tween-20 (Applied Biosystems, Waltham, USA), for a minimum of 1 h. The analysis of protein expression on the arrays was through a catalyzed signal–reporter system. Briefly, the slides were placed in an automated slide stainer (Autostainer; Dako, Carpinteria, USA) and immunostained according to the manufacturer instructions (CSA kit; Dako). A set of specific, validated, antibodies to various phosphorylated or cleaved proteins from different pathways was used to immunostaing the reverse-phase protein microarray. These included: EGFR Y1068; Smad1 (S463/465), Smad5 (S463/465) and Smad8 (S426/428); Akt, Akt S473 and Akt T308; cleaved PARP D214; p38 MAPK T180-182; p70 S6 kinase T389; PKA CT197 and PKC Z-LT410-403; PTEN S380; S6 Rib Prot S235-236; SAPK-JNK T183-185; Src family Y416; Cdk2; Kip 1 P27; Stat3 S727; Creb S133; JAK 1 Y1022-1023; MARKS S152-156; MEK 1-2 S217-221 (Cell Signaling Technology, Beverly, USA); annexins I and II; cyclin B (BD Pharmingen, San Diego, USA); and PRAS 40 T246 (Biosource International, Camarillo, USA). The negative-control slide was incubated with the antibody diluted without the primary antibody. The secondary antibody probes were goat anti-rabbit IgG heavy and light chains (1:5,000 dilution; Vector Laboratories, Burlingame, USA) and goat anti-mouse IgG heavy and light chains (1:5,000 dilution; Zymed Laboratories, San Francisco, USA).

The total protein per microarray spot was determined with the Fast Green FCF protein-blot stain (Sigma), according to the manufacturer instructions, to estimate the total protein amounts for each sample assayed, so as to ensure that the intensity values were not dependent on changes in the concentrations of the assayed lysates. Each array was scanned, the spot intensities were analyzed, the data were normalized to total protein/ spot, and a standardized, single data value was generated for each sample on the array using the Microvigene Software (VigeneTech Inc,), which was specifically developed for reverse-phase protein microarray analysis.

Western blotting was used to confirm the reverse-phase array data. Here, 20 μg protein lysates were loaded onto SDS-PAGE gels, then transferred to nitrocellulose filters (Amersham Hybond-P, GE Healthcare) and assayed for detection with antibodies against the following: Akt, Akt S473, PTEN S380, Src family Y416, Stat3 S727, Creb S133, MARKS S152-156, MEK 1-2 S217-221 and β-actin (1:1,000) (Cell Signaling Technology, Beverly, USA). The secondary antibody was horseradish-peroxidase-conjugated goat anti-rabbit or mouse IgG (1:50,000) (Upstate Biotechnology). The proteins were detected using enhanced chemiluminescence and films (GE Healthcare).

**Tumor-propagating cell analyses and hypoxia conditions**

The proportion of Daoy TPCs was identified through the CD15 and CD 133 cell-surface markers, using a FACS Calibur instrument (Becton Dickinson, San Jose, USA) with antibodies from Milteny Biotec (Auburn, USA), according to the manufacture instructions, with PE-conjugated anti-glycophorin A for CD15, and APC-conjugated-antibodies to CD133. Briefly, the cells were infected with the adenovirus (AdV) as mock and AdV miR-34a, and 36 h later they were plated and cultured in a multi-gas incubator (Sanyo MCO-5M) at different oxygen concentrations (20% for normoxia, 1% for hypoxia) for 12 h. Forty-eight hours after infection, the cells were harvested in Fc receptor blocking reagent and incubated with anti-CD15 and anti-CD133 antibodies for 10 min in the dark at 4 °C. The cells were then washed and resuspended in PBS. Cells expressing higher levels of CD15 and CD133 than the IgG controls were considered positive. At the same time, total RNA was extracted by TRIZOL® (Invitrogen), and 2 μg was retro-transcribed using the ISCRIPT enzyme (Biorad, Pero, Italy), according to the manufacturer protocol. Syber-green real-time PCR was performed using standard protocols, with a 7900-HT FAST real-time PCR sequence-detection system (Applied Biosystems, Foster City, USA), as described previously (Bulfone et al., 2005). Experiments were performed in duplicate, and human β-actin was used as the reference for mRNA expression. The CD15 and CD133 primers were generated using the Primer Express program, from Applied Biosystems.

**Immunohistochemistry**

Unmasking was performed in 10 mM citrate buffer, pH 6, at 97 °C for 45 min. Blocking was performed with the antibody diluent with background reducing components (Dako Cytomation) for 30 min at room temperature; the polyclonal anti-GFAP(1:500), anti-Nestin (1:500) and anti-KI67 (1:300) antibodies were used overnight at 4 °C. The signals were revealed using LSAB DAKO kits for 15 min (biotin, streptavidin) at room temperature. DAB was from DakoCytomation, and the slides were mounted and examined under a DC500 compound microscope (Leica, Nussloch, Germany).

**Animal experiments**

Xenograft subcutaneous implantation. Here, 150,000 viable Daoy miR34 AdV-infected cells and mock-AdV-infected cells were mixed with a 1:1 PBS:matrigel solution (BD Biosciences, San Jose, USA). Six-week-old female athymic mice were anesthetized using Avertin (Sigma), as a 3% solution in tert-amyl alcohol (Fisher), at a dose of 3 mg per 10 g body weight. They were then injected s.c. into each flank with a total volume of 0.1 ml of matrigel PBS cell suspension. To establish intracerebellar xenograft models, 6-10-week-old mice were anesthetized as above, after which the atlanto-occipital membrane was exposed using a small skin incision (5 mm) and a burr hole (0.7 mm diameter) created with a microsurgical drill (Fine Science Tools, Foster City, USA). Daoy-Luc cells (200,000) previously infected at 100 MOI with adenoviral particles carrying an empty vector and pre-miR34 were resuspended in 5 μl PBS and injected slowly through the burr hole into the right cerebellar hemisphere, using a 10-μl, 26-gauge Hamilton gas-tight 1701 syringe needle that was inserted perpendicular to the cranial surface. The wound was sutured with 3-0 polyglactin 910, and 4-0 silk. The mice were imaged after the implantation of the cells, with tumor growth monitored by weekly bioluminescence imaging (BLI) acquisitions using an IVIS 3D Illumina Imaging System (Xenogen/Caliper Alameda, USA). For the acquisitions, the mice were anesthetized with isofluorane i.p., injected with 100 ml D-luciferin (15 mg/ml stock) per 10 g body weight, and 10 min after this luciferin injection, they were imaged for 30 s; four acquisitions were made per mouse (ventral, dorsal and each flank). To quantify the bioluminescence, the integrated fluxes of photons (photons per s) within each area of interest were determined using the Living Images Software Package 3.2 (Xenogen-Caliper, Alameda , USA). From the start of tumor growth, the emission data were collected for at least four weeks, and then they were normalized to the bioluminescence on the injection day. Caliper measurements of tumor sizes were made weekly, along the long and short axes, and estimations of their volumes were made using the formula: volume = width^2^ × length × 0.52. The mice were monitored daily by weight and neurological examinations, to maintain their good standard of health status. Those mice that showing signs of health sufferance were sacrificed immediately. On day 30 after cell inoculation, all of the mice were sacrificed with an overdose of ketanest and xylazin. Tumor samples of the euthanized mice were collected, fixed in 4% paraformaldehyde, and paraffin and OCT embedded for further analyses. The data were analyzed using Excel software or the QuickCalc software (available at [www.graphpad.com/quickcalcs/ttest1.cfm](http://www.graphpad.com/quickcalcs/ttest1.cfm)). For p-value calculations, a two-tailed Mann-Whitney test was used for miR34 expression analyses of the tumor samples, while unpaired two-tailed t-tests were used in all of the other experiments.

**Supplemental literature**

Baek, D., Villen, J., Shin, C., Camargo, F.D., Gygi, S.P., and Bartel, D.P. (2008). The impact of microRNAs on protein output. Nature *455*, 64-71.

Boudreau, R.L., Martins, I., and Davidson, B.L. (2009). Artificial microRNAs as siRNA shuttles: improved safety as compared to shRNAs in vitro and in vivo. Mol Ther *17*, 169-175.

Bulfone, A., Carotenuto, P., Faedo, A., Aglio, V., Garzia, L., Bello, A.M., Basile, A., Andre, A., Cocchia, M., Guardiola, O.*, et al.* (2005). Telencephalic embryonic subtractive sequences: a unique collection of neurodevelopmental genes. J Neurosci *25*, 7586-7600.

Castanotto, D., Sakurai, K., Lingeman, R., Li, H., Shively, L., Aagaard, L., Soifer, H., Gatignol, A., Riggs, A., and Rossi, J.J. (2007). Combinatorial delivery of small interfering RNAs reduces RNAi efficacy by selective incorporation into RISC. Nucleic Acids Res *35*, 5154-5164.

Fan, X., Mikolaenko, I., Elhassan, I., Ni, X., Wang, Y., Ball, D., Brat, D.J., Perry, A., and Eberhart, C.G. (2004). Notch1 and notch2 have opposite effects on embryonal brain tumor growth. Cancer Res *64*, 7787-7793.

Grimm, D., and Kay, M.A. (2007). RNAi and gene therapy: a mutual attraction. Hematology Am Soc Hematol Educ Program, 473-481.

Hallahan, A.R., Pritchard, J.I., Hansen, S., Benson, M., Stoeck, J., Hatton, B.A., Russell, T.L., Ellenbogen, R.G., Bernstein, I.D., Beachy, P.A.*, et al.* (2004). The SmoA1 mouse model reveals that notch signaling is critical for the growth and survival of sonic hedgehog-induced medulloblastomas. Cancer Res *64*, 7794-7800.

Kapsimali, M., Kloosterman, W.P., de Bruijn, E., Rosa, F., Plasterk, R.H., and Wilson, S.W. (2007). MicroRNAs show a wide diversity of expression profiles in the developing and mature central nervous system. Genome Biol *8*, R173.

Krichevsky, A.M., Sonntag, K.C., Isacson, O., and Kosik, K.S. (2006). Specific microRNAs modulate embryonic stem cell-derived neurogenesis. Stem Cells *24*, 857-864.

Li, X.N., Parikh, S., Shu, Q., Jung, H.L., Chow, C.W., Perlaky, L., Leung, H.C., Su, J., Blaney, S., and Lau, C.C. (2004). Phenylbutyrate and phenylacetate induce differentiation and inhibit proliferation of human medulloblastoma cells. Clin Cancer Res *10*, 1150-1159.

Lim, L.P., Lau, N.C., Garrett-Engele, P., Grimson, A., Schelter, J.M., Castle, J., Bartel, D.P., Linsley, P.S., and Johnson, J.M. (2005). Microarray analysis shows that some microRNAs downregulate large numbers of target mRNAs. Nature *433*, 769-773.

Liotta, L.A., Espina, V., Mehta, A.I., Calvert, V., Rosenblatt, K., Geho, D., Munson, P.J., Young, L., Wulfkuhle, J., and Petricoin, E.F., 3rd (2003). Protein microarrays: meeting analytical challenges for clinical applications. Cancer cell *3*, 317-325.

McBride, J.L., Boudreau, R.L., Harper, S.Q., Staber, P.D., Monteys, A.M., Martins, I., Gilmore, B.L., Burstein, H., Peluso, R.W., Polisky, B.*, et al.* (2008). Artificial miRNAs mitigate shRNA-mediated toxicity in the brain: implications for the therapeutic development of RNAi. Proceedings of the National Academy of Sciences of the United States of America *105*, 5868-5873.

Miska, E.A., Alvarez-Saavedra, E., Townsend, M., Yoshii, A., Sestan, N., Rakic, P., Constantine-Paton, M., and Horvitz, H.R. (2004). Microarray analysis of microRNA expression in the developing mammalian brain. Genome Biol *5*, R68.

Paweletz, C.P., Charboneau, L., Bichsel, V.E., Simone, N.L., Chen, T., Gillespie, J.W., Emmert-Buck, M.R., Roth, M.J., Petricoin, I.E., and Liotta, L.A. (2001). Reverse phase protein microarrays which capture disease progression show activation of pro-survival pathways at the cancer invasion front. Oncogene *20*, 1981-1989.

Schwamborn, J.C., Berezikov, E., and Knoblich, J.A. (2009). The TRIM-NHL protein TRIM32 activates microRNAs and prevents self-renewal in mouse neural progenitors. Cell *136*, 913-925.

Selbach, M., Schwanhausser, B., Thierfelder, N., Fang, Z., Khanin, R., and Rajewsky, N. (2008). Widespread changes in protein synthesis induced by microRNAs. Nature *455*, 58-63.

Sempere, L.F., Freemantle, S., Pitha-Rowe, I., Moss, E., Dmitrovsky, E., and Ambros, V. (2004). Expression profiling of mammalian microRNAs uncovers a subset of brain-expressed microRNAs with possible roles in murine and human neuronal differentiation. Genome Biol *5*, R13.

Shen, Y.M., Meltzer, H., Saljooque, F., and U, H.S. (2001). Stimulation of the epidermal growth factor receptor induces glial-specific protein expression in the human DAOY neuroectodermal cell line. Dev Neurosci *23*, 84-90.

Zhao, S., and Liu, M.F. (2009). Mechanisms of microRNA-mediated gene regulation. Sci China C Life Sci *52*, 1111-1116.
